# 3D microstructure design of lithium-ion battery electrodes assisted by X-ray nano-computed tomography and modelling

**DOI:** 10.1038/s41467-020-15811-x

**Published:** 2020-04-29

**Authors:** Xuekun Lu, Antonio Bertei, Donal P. Finegan, Chun Tan, Sohrab R. Daemi, Julia S. Weaving, Kieran B. O’Regan, Thomas M. M. Heenan, Gareth Hinds, Emma Kendrick, Dan J. L. Brett, Paul R. Shearing

**Affiliations:** 1 Electrochemical Innovation Lab, Department of Chemical Engineering, University College London, London, WC1E 7JE UK; 2 National Physical Laboratory, Teddington, Middlesex TW11 0LW UK; 3 The Faraday Institution, Quad One, Harwell Science and Innovation Campus, Didcot, OX11 0RA UK; 4 Department of Civil and Industrial Engineering, University of Pisa, 56122 Pisa, Italy; 5 National Renewable Energy Laboratory, 15013 Denver West Parkway, Golden, CO 80401 USA; 6 School of Metallurgy and Materials, University of Birmingham, Birmingham, B15 2TT UK

**Keywords:** Batteries, Chemical engineering, Imaging techniques

## Abstract

Driving range and fast charge capability of electric vehicles are heavily dependent on the 3D microstructure of lithium-ion batteries (LiBs) and substantial fundamental research is required to optimise electrode design for specific operating conditions. Here we have developed a full microstructure-resolved 3D model using a novel X-ray nano-computed tomography (CT) dual-scan superimposition technique that captures features of the carbon-binder domain. This elucidates how LiB performance is markedly affected by microstructural heterogeneities, particularly under high rate conditions. The elongated shape and wide size distribution of the active particles not only affect the lithium-ion transport but also lead to a heterogeneous current distribution and non-uniform lithiation between particles and along the through-thickness direction. Building on these insights, we propose and compare potential graded-microstructure designs for next-generation battery electrodes. To guide manufacturing of electrode architectures, in-situ X-ray CT is shown to reliably reveal the porosity and tortuosity changes with incremental calendering steps.

## Abstract (web summary)

The 3D microstructure of the electrode predominantly determines the electrochemical performance of Li-ion batteries. Here, the authors show that the microstructural heterogeneities lead to non-uniform Li insertion and current distribution while graded-microstructures improve the performance.

## Introduction

Lithium-ion batteries (LiBs) are the leading energy storage technology for portable electronics and electric vehicles (EVs)^1^, which could alleviate reliance on fossil fuels. However, major challenges in safety, energy and power density, cost and durability of LIBs remain^2–4^: this study aims to provide prospective solutions to these challenges.

The first challenge this work will address is elucidating the optimum electrode design for specific operating conditions. LiB electrodes exhibit complex interplay between multiple electrochemically coupled transport processes, which rely upon the microstructure of the constituent phases and their spatial arrangement^5–7^. Comprehensive understanding of the physical and electrochemical processes at the micro-scale is critical to rationalise the microstructural engineering strategy (e.g. porosity, thickness and mass loading) for different applications^8–10^. For instance, the porosity and pore structure are predominantly determined by calendering process, which effectively reduces electrical resistance and improves energy density of the electrode at the cost of rate capability. However, few studies unravel the dependence of tortuosity and porosity on calendering in real time and whether or not it is appropriate to apply the same calendering strategy to electrodes of different thickness.

The second challenge is to understand the effect of heterogeneous electrochemical, mass transport and mechanical properties, on the long-term durability, safety and electrochemical performance of LIBs^11–13^. Heterogeneous pore phase and particle packing can result in high current densities locally, leading to a non-uniform state-of-lithiation (SoL)^11,14^ and hot-spot formation^13,15^. Consequently, uneven utilisation of the active material as well as non-uniform mechanical (cracking and delamination)^16–18^ and electrochemical (ionic mixing and phase transition) aging^19,20^ significantly reduce the cell’s cycle life, energy density and safety^21,22^, particularly under high-rate conditions^23,24^. Improved understanding of the interplay between microstructural heterogeneities and battery performance not only helps to alleviate degradation, but also provides new insights into advanced structure design.

Mapping the electrode microstructure in 3D is necessary to evaluate microstructural heterogeneities and their effect on battery performance, for which X-ray computed tomography (X-ray CT) has emerged as a valuable technique^17,25^. However, X-ray CT of LiB electrodes is restricted due to the challenge of identifying the carbon-binder domain (CBD). Previously, the pore and CBD were treated as a single phase (pore-CBD phase) that was assumed to be filled with electrolyte^23,26^, which may lead to unrepresentative microstructures and mass transport performance. To accurately correlate the microstructure with performance, a more practical solution is required.

This study aims to drive the advancement of battery performance in the perspectives of both electrode design and manufacturing. Assisted by a novel dual-scan superimposition (DSS) technique that combines the separately imaged low-attenuating CBD and high-attenuating LiNi_1/3_Mn_1/3_Co_1/3_O_2_ positive electrode (NMC111), the full 3D microstructure is reconstructed, metrologically characterised and modelled, by a 3D, microstructure-resolved physics-based battery model of high fidelity, to elucidate the interplay between the microstructure and the electrochemical performance, in order to highlight the effect of heterogeneity on electrochemical-state variables such as SoL, charge transfer, electrolyte concentration and electrochemical potential, both globally and locally. Building on these insights, we propose and compare potential graded-microstructure designs for next-generation Li-ion cells. Finally, in situ X-ray computed tomography is conducted to investigate the microstructural evolution, porosity and tortuosity variation at incremental calendering steps to guide the manufacturing process.

## Results

### 3D characterisation of microstructural heterogeneities

**Fig. 1:**
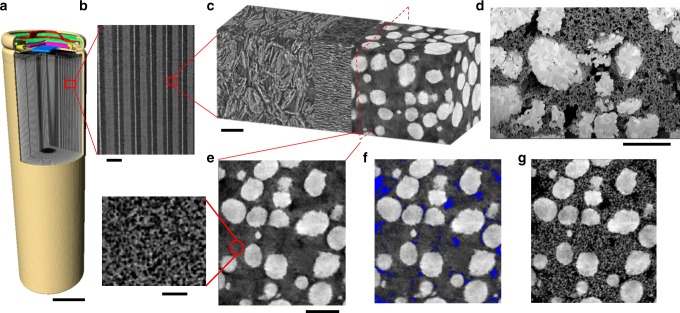
Hierarchical structure of the LiB. **a** Volume rendering of the reconstructed cylindrical battery scanned by X-ray micro-CT (accelerating voltage 180 kV, exposure time 1 s and voxel size 12.9 μm). The metal shell (brown), top button (red), venting disk (green), crimp plate (pink), seal insulator (yellow) and current collector (blue) are shown, with a corner cut displaying the internal structure of the battery; **b** magnified virtual slice to show the periodic layered structure of the cell; **c** X-ray nano-CT image showing (from left to right) the graphite anode, polyolefin separator and NMC cathode; **d** SEM image showing the CBD morphology alongside the secondary NMC particles, wherein the crystallography of the primary particles is seen; **e** virtual slice of the reconstructed 3D electrode, showing a blurry phase comprising the CBD and micro-pores (white: active material particles; dark grey: macro-pores; light grey: blurry mixed phase of micro-pores and CBD). Inset image shows the higher resolution scan of the CBD phase; **f** macro-pores (blue) are highlighted and retained with the active particle in the dual-scan superimposition process; **g** the resultant full microstructure of the NMC cathode by X-ray CT. The scale bars in (**a**, **b**) represent 10 mm and 240 μm respectively, 10 μm in (**c**, **d**, **e**) and 2 μm in the inset of (**e**).

Lithium-ion battery cells are composed of structural constituents spanning over multiple length scales. Figure 1a shows a typical cylindrical LiB scanned by X-ray micro-computed tomography (micro-CT) at a voxel size of 12.9 μm, with a corner-cut-out showing the auxiliary components in the cell cap and the internal structure of the cell. The double-sided coated current collector, cathode, separator and anode are wound round the central supportive core, forming periodic layers in the radial direction (Fig. 1b). X-ray nano-CT was then conducted to capture the 3D microstructural details of the layered cell separately prepared by doctor blade coating method (Fig. 1c). Distinct morphologies in terms of particle shape, orientation and pore size are observed in the graphite anode, polyolefin separator and NMC cathode (from left to right). Further magnification of the cathode microstructure was achieved by SEM imaging (Fig. 1d), in which the microstructural details of the CBD, micro and macro-pores, NMC secondary and primary particles of different orientations are resolved. It is noted that X-ray CT failed to reconstruct and distinguish the microstructure details of the CBD and the micro-pores, which appear as a blurry mixed phase (Fig. 1e), due to the nano-features and the similarly low X-ray attenuation coefficients of carbon and the pores. In addition, the highly X-ray absorbing NMC particles further deteriorate the level of CBD contrast.

In this study, for the first time this challenge has been addressed by a novel dual-scan superimposition technique called DSS to capture the full microstructure of the NMC electrode including the authentic CBD: a second X-ray nano-CT scan of the separate stand-alone CBD sample (i.e. prepared with no NMC particles) with a much higher voxel resolution (63 nm) was undertaken so that the micro-pores inside the blurry phase could be identified due to the enhanced signal-to-noise ratio and better spatial resolution and contrast (inset in Fig. 1e, detailed scanning parameters in Methods section). By retaining the NMC particles and macro-pores (labelled as blue in Fig. 1f) in the first scan and replacing the blurry mixed phase with the reconstructed second scan, the fully representative electrode microstructure is obtained (Fig. 1g), which corresponds well to the SEM image (Fig. 1d) in terms of porosity and pore size distribution.

**Fig. 2:**
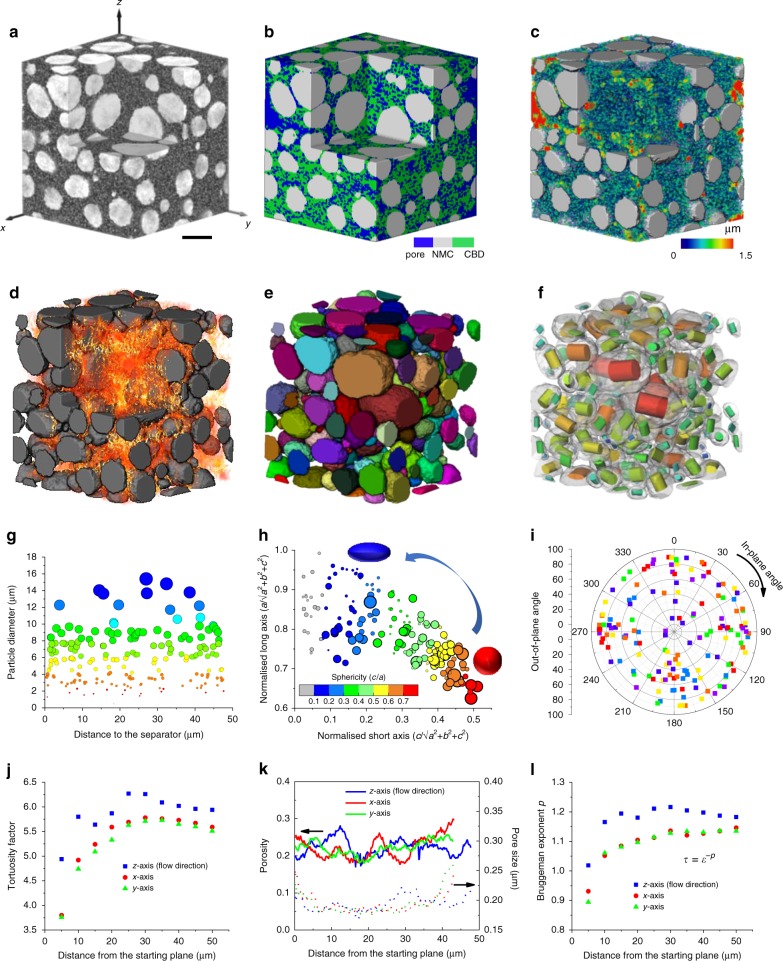
3D characterisation of the NMC cathode based on X-ray CT data. **a** Reconstructed volume of the electrode with different phases represented by greyscale values (white: NMC active material; dark grey: CBD; black: pore). The corner is cut to show the internal morphology; **b** segmented three phases; **c** colour-coded pore phase according to its size, shown with NMC particles; **d** Li^+^ ion diffusive flux map in the pore phase used for effective mass transport estimation; **e** identification of individual NMC particles; **f** visualisation of the shape, orientation and size of each individual particle; **g** resultant particle size distribution as a function of distance from the separator. Symbols are colour-coded according to the particle size; **h** sphericity distribution of the NMC particles. The size of the scatter points represents the size of the particles and the colour codes represent the sphericities; **i** orientation map of the individual particles, with the azimuthal angles and radial distance representing the in-plane and out-of-plane orientations; **j** tortuosity factor obtained based on the diffusive flux from (**d**) in three directions; **k** slice-by-slice variation of porosity and pore size in three orthogonal directions; **l** Bruggeman exponents as a function of the fraction of the electrode thickness in three orthogonal directions. The scale bar in (**a**) represents 10 μm.

Figure 2a shows the reconstructed cathode with the three phases represented by greyscale values that are proportional to their respective X-ray attenuation coefficients, based on which the three phases are segmented (Fig. 2b) and a heterogeneous pore phase is observed. Figure 2c displays the colour-coded spatial distribution of pore size, whose heterogeneity correlates to the simulated Li-ion diffusive flux (Fig. 2d, simulation details in Supplementary Eq. 14 and Supplementary Fig. 2): the heterogeneity of the pore size results in locally high flux (i.e. bright areas) and could lead to regions of higher ionic transport resistance and elevated temperature. Figure 2e identifies each individual NMC particle, with the shape and orientation visualised in Fig. 2f. The particle size as a function of distance from the separator is plotted in Fig. 2g, showing a uniform distribution of the particle size along the through-thickness direction (perpendicular to the current collector) and a wide scattering (between 2 and 15 μm) at each depth of the electrode. Figure 2h shows that larger particles are more spherical than smaller ones. An extremely broad sphericity distribution is observed, calling into question the validity of cathode models based on ideal spherical particle geometry. It is worth mentioning that the sphericity of the active particles has been observed to decrease during long-term cycling as a consequence of phase transformation and stress accumulation^27^. The orientation distribution of these particles is random (Fig. 2i). The volume-averaged tortuosity factor τ, which is often used to describe the effective conductivity/diffusivity of the pore phase, increases significantly over the first 30 μm (four times the average particle size) from the starting plane (Fig. 2j), highlighting the inhomogeneous microstructure. The porosity displays severe fluctuation, which could arise from the local variation of the solid particle distribution (Fig. 2k), as supported by a good correspondence between the local porosity (blue curve in Fig. 2k) and the particle distribution in Fig. 2g (region 10−20 μm from the separator). These are all microstructural heterogeneities that should be considered in electrode design, manufacturing and battery modelling. Figure 2l plots Bruggeman exponents (*p* in *τ* = *ϕ*^−^^*p*^, *ϕ* is the porosity) that increase from approx. 0.9 to 1.15, significantly higher than the empirical value for spherical particles (*p* = 0.5).

### Microstructure and performance correlation

**Fig. 3:**
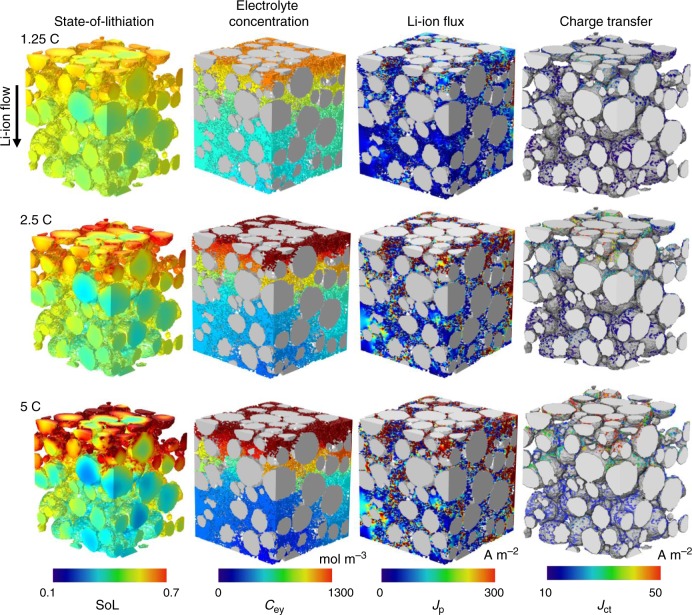
Simulated discharge of the reconstructed cathode and an ideal lithium anode (half-cell) at 1.25, 2.5 and 5 C. The ionic current flows from the top (separator side) to the bottom (current collector side). Field variables at 50% depth-of-discharge (DoD) are shown here.

To understand the interplay between battery microstructure and performance, dynamic discharging processes (i.e. cathode lithiation) at different C-rates were simulated using the reconstructed cathode microstructure and an ideal metallic lithium anode (Fig. 3). The illustration of the physical processes, mathematics and parameters is shown in Supplementary Fig. 4 and Supplementary Table 1−3. The simulated voltage vs. capacity curves are compared with the experimental data of the fabricated coin cells (NMC111 vs. lithium) at different C-rates (Supplementary Fig. 6). Figure 3 shows that the SoL is relatively uniform at 1.25 C, while a gradient starts to develop at 2.5 C and becomes severe at 5 C. This is consistent with the increasing gradient along through-thickness direction of electrolyte salt concentration *C*_ey_ in the second column. The competition between reaction kinetics and mass transport dynamics determines the SoL and *C*_ey_ gradients: at high C-rates the reaction is relatively fast so that the sluggish mass transport in the convoluted 3D pore phase cannot supply the reactant (Li^+^ ion) deep into the electrode at a sufficient rate. Consequently, the charge transfer predominantly occurs near the separator where *C*_ey_ is high, while the NMC particles that are further away are not fully utilised leading to severe capacity underutilisation. Furthermore, more homogeneous SoL of intra- and inter-particles is observed at 1.25 C, compared to the higher C-rates wherein significant disparity of the SoL is evident between small and large particles and also from the core to the surface of the large particles, as a consequence of the ratio between the solid-state diffusion and reaction kinetics. The heterogeneous SoL inevitably creates temperature gradients and thermal/mechanical stress^28,29^ both locally in individual particles and between neighbouring particles, leading to cracking and delamination that reduce the battery lifetime^15^.

The third column displays the distribution of Li^+^ ion flux *J*_p_. At 1.25 C the flux is evenly distributed; however, significant heterogeneity is observed beyond 2.5 C due to the non-uniform pore microstructure, in which the Li^+^ ion flux is much higher in narrower pores or in low porosity regions. This would cause local Ohmic heating, potentially giving rise to electrolyte decomposition^30^ and initiating thermal runaway^13^. The final column shows the charge transfer *J*_ct_ (i.e. reactivity) distribution at the electrolyte/particle interface, which is observed to be more homogeneous at low C-rates whereas at high C-rates, reactivity variation is observed particle-by-particle as a consequence of local microstructural heterogeneity: regions with higher current densities are expected to degrade faster than average. In addition, due to the mass transport limitation, *J*_ct_ is significantly higher in the vicinity of the separator than the current collector at high C-rates, yielding the “switch-off” of substantial reaction active sites.

**Fig. 4:**
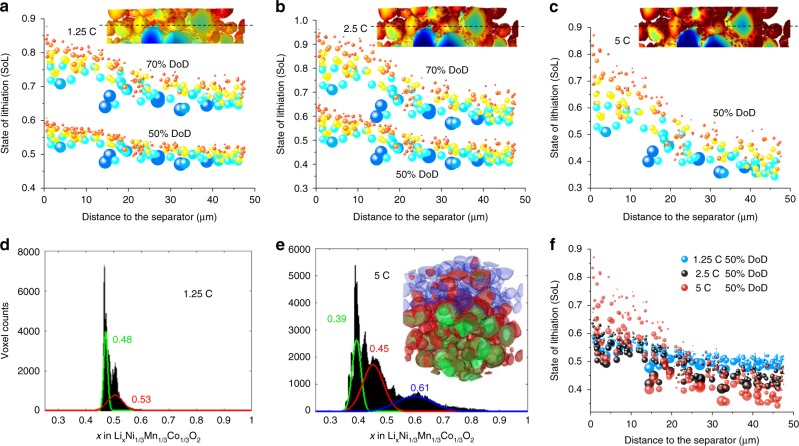
Analysis of particle-by-particle variation of SoL under different operating conditions. The colour of the symbols corresponds to the particle size (blue to red: large to small) to enhance the visibility, and size of the symbols is proportional to the particle size. At 5 C, 70% DoD is not accessible so only 50% is shown. The insets in (**a**−**c**) compare the different degrees of heterogeneous SoL in the same region (15 μm from the separator) as a function of discharge rate. **d** and **e** compare the histogram of SoL in the active material at discharge rates of 1.25 and 5 C where the SoL values of main peaks are identified with different colours. The particles in the 3D rendering in (**e**) are colour-coded according to the three peaks in the histogram, and **f** comparison of SoL of the corresponding individual particles at three discharge rates.

Analysis of the SoL in each particle in the through-thickness direction at different C-rates was conducted (Fig. 4). As the discharge rate increases, the impact of particle size on SoL heterogeneity becomes more pronounced: at 5 C significant heterogeneity is observed both transversely and longitudinally along the through-thickness direction, with up to 60% variation in SoL between small and large particles close to the separator (Fig. 4c). The insets in Fig. 4a−c compare the uniformity of lithium concentration at three discharge rates at 50% depth-of-discharge (DoD). The histogram in Fig. 4d shows the macroscopically narrow distribution of SoL at 50% DoD, 1.25 C, with two distinct peaks, representing a more uniform particle lithiation compared to Fig. 4e, which displays a broader SoL distribution at 5 C. The three distinguishable peaks are more separated (i.e. incrementally severe lithiation separation) as a function of discharge time (Supplementary Fig. 7), which is consistent with the electrochemical Biot number plot (Supplementary Fig. 8) that characterises the relative rates between solid-state diffusion and interfacial reaction kinetics. The inset in Fig. 4e visualises the particles corresponding to the three peak regimes and correlates SoL with particle size and depth. Figure 4f compares the SoL at the three discharge rates, highlighting how the heterogeneous SoL builds up locally and along the through-thickness direction as C-rate increases. In summary, the inhomogeneous lithiation is mainly due to the variation in lithium ion diffusion path associated with the broad distribution of particle size. The uneven utilisation of the NMC particles inevitably leads to a lower energy density than the theoretical one, and likely undermines the long-term structural integrity of the electrode.

**Fig. 5:**
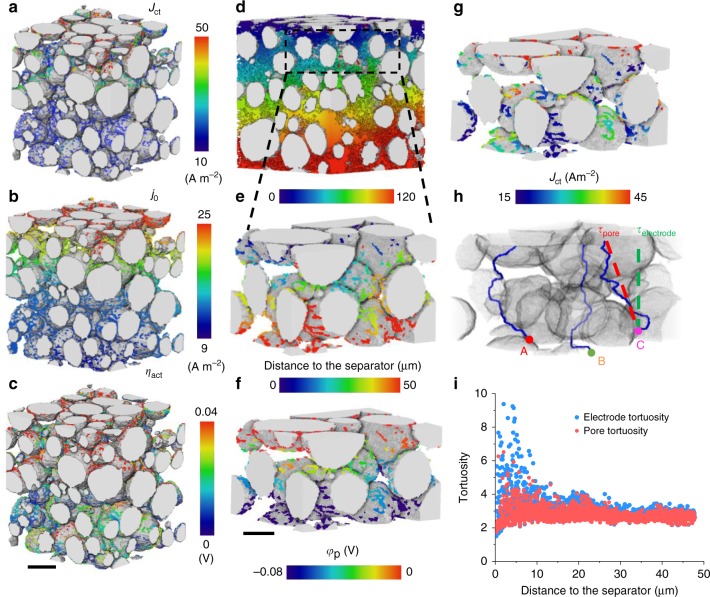
Heterogeneous charge transfer analysis. Spatial distribution of **a** charge transfer current density *J*_ct_; **b** exchange current density *j*_0_; **c** activation overpotential *η*_act_; **d** distance map of every single voxel in the pore phase to the nearest electrode/separator interface measured by a 3D fast marching algorithm; **e** travel distance of the lithium ion to the reaction sites is well correlated with **f** the potential drop in the electrolyte (*φ*_p_), which is consistent with **g** the local variation of charge transfer current density *J*_ct_. **h** Illustration of the tortuosity in the pore phase *τ*_pore_ and electrode *τ*_electrode_ respectively. A, B and C exemplify three distinctly convoluted pathways for Li^+^ ion flow; **i** plot of *τ*_electrode_ and *τ*_pore_ as a function of distance from the separator. The scale bars in (**c**) and (**f**) represent 10 and 7 μm, respectively.

The heterogeneous SoL is also a consequence of non-uniform current density distribution. Figure 5a exhibits the distribution of charge transfer current density *J*_ct_ at the NMC particle/electrolyte interface at 5 C. The spatial distribution of exchange current density *j*_0_ and activation overpotential *η*_act_ are displayed in Fig. 5b and c respectively, which are linked to *J*_ct_ via the Butler−Volmer equation (Supplementary Eq. 11). The *η*_act_ is in good agreement with the experimentally measured values^31^. 2D histogram correlation is used to analyse the spatial co-localisation of *J*_ct_ with *j*_0_ and *η*_act_ respectively, concluding that the non-uniform *J*_ct_ is strongly correlated with *η*_act_ rather than *j*_0_ (Supplementary Fig. 9). This is reasonable considering that *J*_ct_ is exponentially dependent on *η*_act_, which is further decomposed to equilibrium potential *V*_eq_ and electrolyte potential *φ*_p_ at the reaction interface (Supplementary Fig. 9e−f). In view of the larger local variation of *φ*_p_ (Δ*φ*_p_ = 0.080 V) compared to *V*_eq_ (Δ*V*_eq_ = 0.025 V), which is essentially dependent on SoL, we conclude that *η*_act_ is mainly governed by *φ*_p_ (more discussion in Supplementary Note 5). Thus, it is necessary to determine the cause of heterogeneity in *φ*_p_ to understand the non-uniform current distribution.

Since Ohm’s law governs ion migration in the electrolyte^32^, the local value of *φ*_p_ is determined by the potential drop in the electrolyte, associated with transport distance from the separator (Fig. 5d). Although the electrode thickness is 50 μm, the actual travel distance for the pore close to the current collector is more than twice this value, implying a substantial impact of convoluted 3D trajectory on mass transport. A magnified region from Fig. 5d shows the local variation of travel distance of lithium ions to the interfacial reacting site (Fig. 5e), which turns out to be inversely correlated with the potential distribution in the electrolyte *φ*_p_ (Fig. 5f), consistent with the heterogeneous charge transfer current density (Fig. 5g). Therefore, we can attribute the local variation of reaction activity to the microstructural heterogeneity which causes the local discrepancy of *φ*_p_. This is further supported by analysis of local tortuosity at each individual reaction site, which can provide more geometrical insights than the widely used macro-homogeneous tortuosity factor^33,34^. Two types of tortuosity are characterised (Fig. 5h): (1) *τ*_pore_, the ratio of the shortest travel distance (blue trajectory) to the Euclidean distance (red dashed line) from the top plane to the reaction site, representing the resistance associated exclusively with the pore phase; (2) *τ*_electrode_, calculated by dividing the shortest travel distance by the vertical distance (green dashed line) to the top plane, delineating the tortuous pathway as a result of both the pore phase and particle morphology.

Figure 5i plots tortuosity vs. distance from the separator for all reaction sites. There is a sharp decrease in *τ*_electrode_ within 15 μm of the separator/electrode interface, with a large scatter followed by a plateau in which *τ*_electrode_ converges to 3. *τ*_pore_ exhibits a lower gradient than *τ*_electrode_ close to the separator and converges to the same level as *τ*_electrode_ after 15 μm. This further emphasises the contribution of particle size and shape to the transport resistance of lithium ions close to the separator, where the particle morphology dominates the ion transport path. As the distance from the separator increases, the impact of particle size and elongation decreases, thus *τ*_electrode_ and *τ*_pore_ reach similar values close to the current collector. This also implies that, at low C-rates when the battery performance is not mass-transport limited, a volume-averaged tortuosity factor can be safely used for model prediction. However, at high C-rates, the performance is heavily dependent on mass transport properties which are primarily determined by particle size and shape close to the separator. Under these circumstances, using a volume-averaged tortuosity factor will inevitably lead to an over-estimation of the lithium-ion flux which can propagate into the parameterisation process for battery management systems (BMSs).

### Design and optimisation of next-generation electrodes

**Fig. 6:**
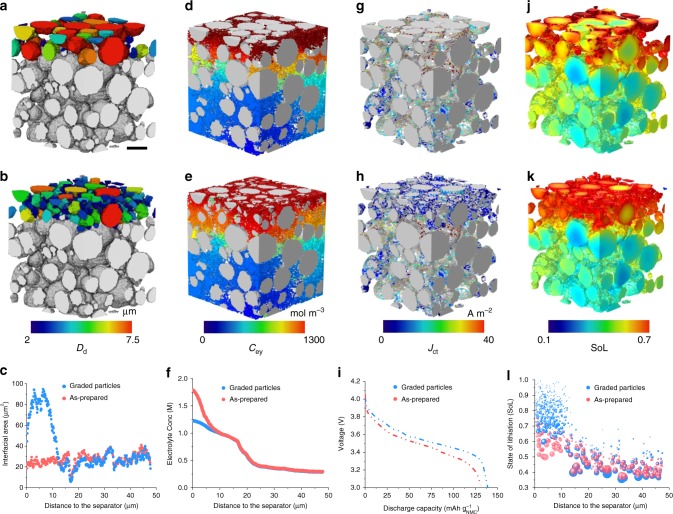
Comparison between the as-prepared (top row) and graded-particle (middle row) samples at 50% DoD and 3.75 C discharge. **a**, **b** Comparison of the particle size distribution; **c** change in interfacial reaction area as a function of the depth in the electrode; **d**−**f** comparison of the distribution of the electrolyte concentration; **g**, **h** distribution of charge transfer current density and **i** the resultant voltage vs. capacity curve; **j**−**l** comparisons of the SoL. The scale bar in (**a**) represents 10 μm.

The above insights can be applied to the design of improved graded-microstructure electrodes, which have recently attracted increasing attention^35,36^. Compared with the as-prepared microstructure (Fig. 6a), here we obtain the graded-particle structure (Fig. 6b) by morphological erosion^37,38^ of the initial particles in a region within 12 μm of the separator/electrode interface and filling the gap between particles with small ones so as to maintain the same active material volume fraction (see Methods for more details). The decrease of particle size benefits the electrochemical performance by increasing the number of interfacial reaction sites (Fig. 6c), thereby enhancing the surface exchange kinetics. The electrochemical performance is compared at 50% DoD and 3.75 C discharge. The second column in Fig. 6 shows that in the fine-particle zone the electrolyte distribution is more uniform, associated with the reduced Li^+^ ion transport resistance due to the particle geometry. The third column evidences a significant homogenous *J*_ct_ distribution in the graded-particle sample (Fig. 6h) compared to the as-prepared one (Fig. 6g), which arises from the higher specific surface area and more uniform potential distribution in the electrolyte, contributing to an increased discharge capacity and average voltage (Fig. 6i). The graded-particle structure provides more homogeneous lithiation and better utilisation of active material (Fig. 6k vs. Fig. 6j and Fig. 6l).

**Fig. 7:**
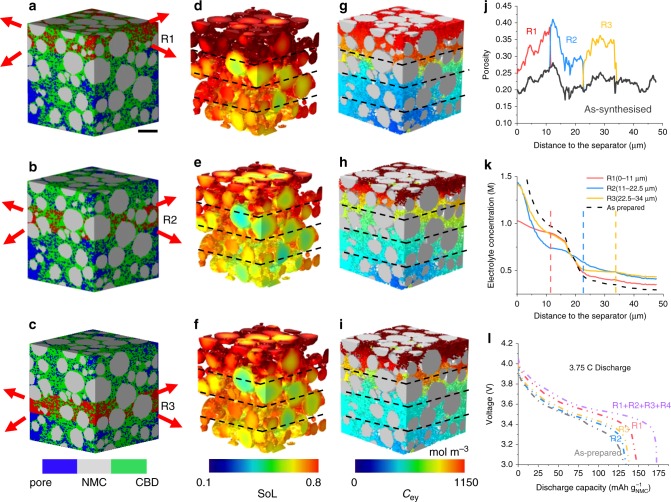
Investigation of LiB electrode design with graded-porosity/pore size. Pore microstructure is enlarged locally by 250 nm: **a** 11 μm (R1), **b** 22.5 μm (R2), and **c** 34 μm (R3) away from the separator. The processed regions are highlighted in red; **d**−**f** maximum accessible SoL comparison between the three samples (84%, 77% and 76% DoD) discharged at 3.75 C, with the dashed lines (also those in (**k**)) representing the boundaries of the three processed zones; **g**−**i** the corresponding distribution of electrolyte concentration; **j** plot of porosity vs. electrode thickness for the three samples, labelled as R1, R2 and R3, with the grey curve representing the baseline for the as-prepared sample; **k** local concentration gradient suppressed by the enlarged pores in each region at 50% DoD; **l** comparison of electrochemical performance for different microstructural designs. R1 + R2 + R3 + R4 refers to global dilation of the pore microstructure. The scale bar in (**a**) represents 10 μm.

The electrode electrochemical performance is closely related to mass transport capability, especially close to the separator for high C-rates. To investigate this, morphological dilation^37,38^ of the local pore structure (see Methods for more details) was conducted to enlarge the pore size by 250 nm at three distances away from the separator to obtain graded-pore structures (R1, R2 and R3 in Fig. 7a−c). The porosity increases by shrinking the CBD, while the NMC particles are unchanged. In Fig. 7j, the black curve represents the original porosity as a function of the depth in the as-prepared sample, while the porosity in the modified regions is labelled as R1, R2 and R3. In this way, the contribution of the pores to the global electrochemical performance can be decoupled from the particle microstructure, which is not possible experimentally due to sample-to-sample variations.

Figure 7d−f compare the SoL between the three samples at the end of discharge process at 3.75 C. Sample R1 significantly outperforms the other two. Combined with Fig. 7k, each sample shows respectively the lowest gradient of electrolyte concentration in the corresponding processed region (Fig. 7g−i), due to the locally enhanced mass transport, as the graded-particle electrode demonstrated previously. Although Samples R2 and R3 exhibit similar performance, the locally boosted mass transport still yields a better performance compared to the as-prepared electrode (Fig. 7l). Sample R1 shows a considerable increase in the accessible capacity. It is noted that the rise of local porosity not only enhances the mass transport but is also accompanied by an increase of active particle/electrolyte interface. As the C-rate increases, the reaction active region resides closer to the separator, where R1 provides the most electrochemically favourable conditions compared to R2 and R3. A high porosity sample is also added for comparison (i.e. R1 + R2 + R3 + R4, porosity approx. 33%), displaying 16% more capacity than R1 with a similar average voltage. These results indicate that the battery electrochemical performance is predominantly governed by the porosity at the separator/electrode interface at high C-rates, proving the efficacy of graded-structure electrode design.

**Fig. 8:**
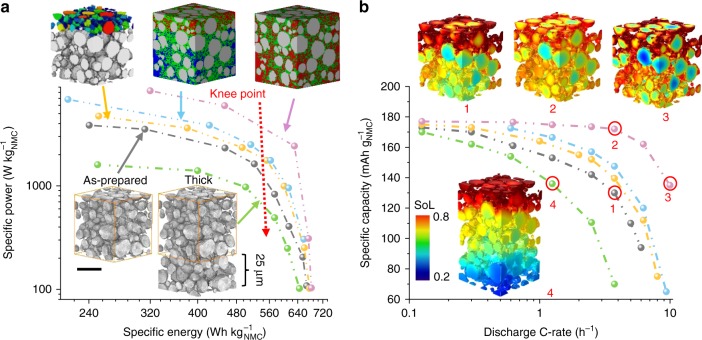
Comparison of the simulated electrochemical performance for different electrode designs. **a** Ragone plot comparing the specific power vs. energy for a variety of electrode microstructures in lithium metal half-cell (grey: 50 μm electrode; green: 75 μm electrode; yellow: graded particle; light blue: graded pore; pink: high porosity). The specific energy is obtained by integrating the discharge curve to the cut-off voltage (3 V) and the specific power is obtained by dividing the specific energy with the total discharge time; **b** maximum accessible specific capacity as a function of C-rate for different microstructures. The inset figures compare the spatial distribution of SoL under different conditions, corresponding to the numbers on the curve respectively. The scale bar in (**a**) represents 20 μm.

The performance of each electrode design is summarised using the Ragone plot in Fig. 8a. Regarding the as-prepared electrode as a baseline, there is great possibility to improve the battery performance using different strategies. In this study the increase in porosity was achieved by pushing the pore/CBD boundary towards the CBD while maintaining the voxels constituting the active material unchanged so that the NMC mass loading is constant. At the porosity level studied, no obvious polarisation arose from the electron-conducting phase (CBD and NMC); however, with further increase in porosity, the connectivity of electron-conducting pathways could be reduced, undermining the accessible specific energy, which will be investigated further in the next section. Thus, a homogeneous distribution of the solid materials is critical in pursuing high porosity microstructures.

For LiBs designed for hybrid applications, the graded-pore structure (light blue curve in Fig. 8) will be a prospective choice for microstructural optimisation as it provides considerable enhancement of both specific energy and power. This structure can be achieved by the addition of pore-formers or other advanced techniques such as ice-templating^39,40^. Since the porosity in the vicinity of the current collector is not very important at high C-rates, it is advised to maintain a high mass loading in this region instead of sacrificing volumetric energy density available at low C-rates for only a modest increase in power density at high C-rates.

For applications prioritising the maximum stored energy, large area-specific mass loading (g cm^−2^) is the most common choice. In Fig. 8a, a cathode of 75 μm thickness (green curve) suffers from loss of power density. In addition, the thicker electrode shows inferior specific energy, deteriorating significantly with the increase of electrical load, as also proved by experimental data^41^. Given the trade-off between specific energy and power, a critical “knee point” should be noted on each curve (dashed red arrow). This point represents the highest accessible specific power before a catastrophic drop of specific energy, which is often integrated in BMSs to maximise the energy output while ensuring battery durability. Apart from the presented designs, extended studies can be conducted to examine the influence of materials processing on the specific power and energy.

Figure 8b compares the specific accessible capacity of these designs as a function of the C-rate. All samples manifest almost identical accessible specific capacity at very low C-rate (0.125 C), but the capacity falls in a distinct manner with increasing C-rate as a consequence of the increasing polarisation of reactant transport and reaction kinetics. Both of the graded-structures show substantial improvement compared to the as-prepared electrode (grey curve), especially at higher C-rates. The spatial distribution of SoL for the as-prepared (Volume 1), high porosity (Volumes 2 and 3) and thick electrodes (Volume 4) at 60% DoD is presented alongside the discharge curves. Comparing Volumes 1 and 2, the enlarged pore size assists a more uniform lithiation along the through-thickness direction; however, intra-particle heterogeneity still exists even in the high porosity sample, implying a limiting effect of solid-state diffusion. Volume 3 exhibits a more severe intra- and inter-particle difference of SoL due to the exacerbated competition between solid-state diffusion and surface exchange rate at 10 C compared to Volume 2 (3.75 C, the onset of Li^+^ ion transport limitation). Volume 4 also shows a wide distribution of SoL even at 1.25 C, highlighting the predominant resistance from mass transport, which may be reduced by enhancing the cationic transference number in the electrolyte^24^.

So far in this study, the influence of microstructural heterogeneity (particle and pore size distribution, spatial arrangements and local mass transport property) on the energy and power density, the efficacy of graded-structure and a comprehensive comparison of different electrode microstructures have been investigated. The image-based modelling technique has been shown as a promising tool for advanced microstructural design and optimisation. To better convert the improved knowledge acquired in the section above to battery engineering, the next section will introduce the application of image-based modelling and DSS technique in rationalising the manufacturing protocols for desired microstructures.

### Time-lapse X-ray nano-CT to track microstructural evolution during calendering

**Fig. 9:**
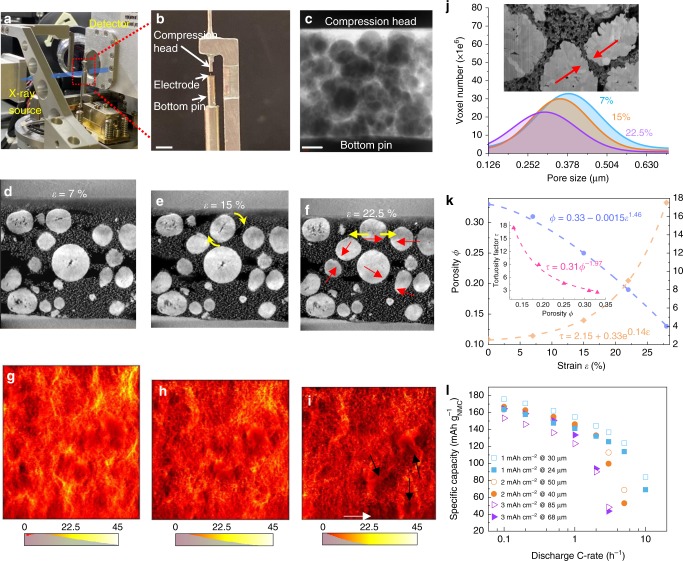
Calendering effect on electrode microstructure and performance. **a** Experimental setup of the time-lapse X-ray nano-CT; **b** magnified image showing the details of the alignment; **c** X-ray radiograph before the compression; **d**−**f** slices from nano-CT images showing the electrode microstructural evolution under incremental calendering steps; **g**−**i** simulated mass flux map in the calendered pore structures respectively, with the colour legend overlaid by the flux histogram; **j** pore size distribution at incremental calendering steps. The inset displays the ‘crushed’ CBD and loss of porosity; **k** quantification of the microstructural parameters as a function of calendering steps based on the reconstructed 3D volumes; **l** experimental electrochemical performance comparison between electrodes of three mass loadings, calendered and uncalendered. The scale bar in (**b**, **c**) represents 2 mm and 10 μm respectively.

A time-lapse X-ray nano-CT experiment was conducted to track the microstructural evolution of the electrode under incremental calendering steps. A novel nano-mechanical test stage was developed for integration into the Zeiss Xradia Ultra series X-ray microscopes^42^. The assembly and experimental setup are shown in Fig. 9a while Fig. 9b shows the alignment of the electrode pillar (65 μm) under the compression head. Figure 9c is an X-ray radiographic projection showing the positioning of each component. Tomographic slices of three different compression steps are displayed in Fig. 9d−f. From compressive strain level *ε* = 7% to *ε* = 15%, an NMC particle is observed to roll against the adjacent ones (yellow arrows). Both the macro and micro-pores decrease in size. With the increase of compression (*ε* = 22.5 %), apart from the further decrease in porosity and pore size, the free-moving particle is constrained by the surroundings and deforms significantly in the horizontal direction, which results in a more compacted structure (red arrows). Figure 9g−i displays the 2D-projected mass flux map from the image-based computed fluid dynamics (CFD) simulation on the calendered 3D pore structure at respective compression levels, with the colour legend overlaid by the histogram. It is noted that the global intensity of the mass flux drops as a consequence of the loss of porosity. The localised high flux region (yellow) diminishes as well, reflecting the closure of narrow pores. It is concluded that when the calendering strain is above 20%, the electrode becomes more compacted and significant loss of porosity could be found locally, either close to the current collector (white arrow in Fig. 9i) or in the vicinity of the active particles (black arrow). Figure 9j and k summarises the variation of the pore size distribution and the structural parameters of the electrode under the incremental calendering steps, which can not only be used to predict the tortuosity based on porosity and therefore guide electrode manufacture, but is also regarded as a reliable parameterisation to be used in reduced-order models for BMSs. The inset in Fig. 9j displays the ‘crushed’ CBD between adjacent particles and the consequent loss of reaction sites.

Additional insights into electrode engineering come from the analysis of how the same calendering strain affects electrodes with different mass loadings. Three mass loading electrodes (1, 2 and 3 mAh cm^−^^2^) were prepared both uncalendered (30, 50, 85 ± 0.8 μm) and calendered at 20% compression (24, 40, 68 ± 0.6 μm) which were subsequently electrochemically cycled at different C-rates (Fig. 9i). Three repeated tests were carried out on each type of the electrodes. The same calendering strain affected the electrodes with different mass loadings distinctly: for the low mass loading electrode higher specific capacity was obtained without calendering, while the opposite applied to the high mass loading electrode (Fig. 9i). These opposite trends are rationalised by the insights gained in the previous sections on electrode heterogeneity: (1) in the low mass loading electrode, calendering exacerbates the microstructural heterogeneity as compression can ‘switch-off’ a substantial number of electrochemical-reactive sites due to the porosity decrease (red arrows in Supplementary Fig. 10), leading to capacity loss regardless of the C-rate. This suggests that the particle size used (*D*_50_ ≈ 5 μm and *D*_90_ ≈ 10 μm; see Supplementary Fig. 11) is too large to provide sufficient specific and total NMC particle/electrolyte interfacial area, consequently incurring inhomogeneous reaction and capacity underutilisation. The general trend of rate capability, though, is identical in the calendered and uncalendered electrodes, indicating that Li^+^ ion transport is not rate-limiting; (2) the calendered high mass loading electrode performs better than the uncalendered one at low C-rate, while almost identical specific capacity is obtained at high C-rate. This is attributed to the long-range electrical percolation and contact that calendering promotes, which is significant at low C-rate when intercalation occurs within the whole electrode volume, while it becomes irrelevant at high C-rate, when reaction takes place close to the separator due to Li^+^ ion transport limitation in the electrolyte (as was observed in Fig. 3). Notably, the experimental curve is consistent with the predicted performance of the thick electrode in Fig. 8b; (3) for the intermediate mass loading, the capacity is identical between calendered and uncalendered samples until reaching 2 C, after which the rate capability loss due to compression becomes significant.

In summary, as manufacturing guidelines, calendering would not benefit the volumetric energy density of power-oriented low mass loading thin electrodes (1 mAh cm^-2^) due to the significant microstructural and reaction heterogeneity that is mainly caused by the large particle size; 20% calendering is feasible to improve the volumetric energy density with moderate rate capability for hybrid applications; higher calendering is recommended for energy-oriented cells to enhance the volumetric energy density further, at the cost of catastrophic capacity drop beyond 1 C. This unique insight into the manufacturing process is only possible with the new advances in imaging, and image-based modelling presented for the first time here.

## Discussion

In this study, we addressed critical technical challenges both in advanced electrode design and engineering for different applications, assisted by the combination of X-ray CT-based techniques and experiments. The DSS technique that combines separate scans of high-attenuating NMC and low-attenuating CBD enables us to reconstruct the 3D electrode including microstructural heterogeneities at nanoscale for the first time. The 3D microstructural-resolved electrochemical model highlighted that, macroscopically, the gradients of electrolyte concentration and SoL increase with C-rate and depth-of-discharge in the electrode through-thickness direction due to Li^+^ ion transport limitation in the electrolyte. The heterogeneous particle and pore phase distribution lead to non-uniform reactivity and reactant transport, inducing uneven intercalation behaviour between particles, which causes underutilisation of capacity and reduced power density, particularly under high rate conditions. Graded-microstructure electrodes are shown to significantly improve the rate capability while maximising the accessible energy density at high mass loading as the porosity in the vicinity of the separator predominantly determines the high rate performance. Thicker electrodes show more reduced specific capacity due to mass transport limitations. Calendering has opposite effects on thin and thick electrodes: while it would not benefit the volumetric energy density of power-oriented thin electrodes due to the significant heterogeneity, 20% calendering can improve the energy density with moderate rate capability for hybrid applications and higher calendering is recommended for energy-oriented cells, although at the cost of catastrophic capacity drop beyond 1 C. The DSS method and modelling techniques presented in this study are applicable across multiple length scales to many other advanced energy conversion and storage devices, such as supercapacitors and fuel cells, to provide new insights into the strategy of electrode design and optimisation.

## Methods

### Sample preparation and coin cell assembly

A mixture of 90 wt% LiNi_1/3_Mn_1/3_Co_1/3_O_2_ (NMC111) (Targray, Canada), 5 wt% conductive carbon black (Timical Super C65, Imerys, Switzerland) and 5 wt% polyvinylidene fluoride (PVDF) (Arkema, France) was homogenised in a dual asymmetric centrifuge system (SpeedMixer DAC 150.1 FVZ-K, Hauschild, Germany). *N*-methyl-2-pyrrolidinone (NMP; anhydrous, Sigma-Aldrich) was used as a solvent. The slurry was cast onto a 20-µm-thick aluminium sheet and spread with a doctor blade. The electrode sheet was then initially dried in an oven prior to further drying in a vacuum oven overnight at 80 °C. The stand-alone CBD sample was prepared by mixing and homogenising 50 wt% conductive carbon black and 50 wt% PVDF with NMP as the solvent in an asymmetric centrifuge system, followed by casting and drying in the vacuum oven overnight at 80 °C. Electrode sheets with thicknesses of 50 μm (1.4 mAh cm^−2^) and 100 μm (2.8 mAh cm^−2^) were prepared. The 50-μm-thick NMC cathode was punched to a diameter of 15 mm and assembled in coin cells (type CR2032) with a lithium anode and a Celgard 2325 separator under a protective argon atmosphere. One hundred microlitres of 1 M LiPF_6_ in EC/EMC (3:7 v/v) was used as the electrolyte.

### Electrochemical testing

Discharge tests were performed on the assembled coin cells (three repeat tests) at room temperature using a potentiostat (Interface 1000E, Gamry Instruments or Maccor 4300) from 4.25 to 3 V. The OCV of NMC111 vs. Li was measured using the Galvanostatic Intermittent Titration Technique (GITT, Supplementary Fig. 5). The coin cells were discharged at a constant current corresponding to a C-rate of C/10 in steps of 10% depth-of-discharge, followed by a pause at open circuit for 5 h to allow the voltage to relax prior to OCV measurement. This pulse/relaxation cycle was repeated until the 3 V cut-off voltage was reached.

### X-ray computed tomography and 3D image analysis

An electrode disk of approximately 1 mm in diameter was punched from the same sheet of cathode material as described above, and then was mounted on a pin head using fast-setting epoxy. The electrode disk was reduced to a pillar of ca. 90 μm in diameter using a micro-milling laser technique (A Series/Compact Laser Micromachining System, Oxford Lasers, Oxford, UK). The electrode pillar was then scanned using a lab-based X-ray nano-CT system (Zeiss Xradia Ultra 810 X-ray microscope, Carl Zeiss, CA, USA)^43^ at an isotropic voxel size of 126 nm and a field of view of 64 μm × 64 μm. A quasi-monochromatic X-ray beam with a Cr characteristic emission energy of 5.4 keV was used and 2001 sequential projections with an exposure time of 14 s were collected over 180° rotation. For the CBD imaging, a voxel size of 63 nm and exposure time of 40 s were used and the other parameters remained the same. The radiographic projections were automatically reference corrected, followed by the alignment of centre shifts (post collection). The corrected, aligned radiographs were reconstructed using standard, parallel beam, filtered-back projection algorithms^44^, implemented in the Zeiss Scout and Scan software package (Carl Zeiss, CA, USA). The reconstructed 3D volume of the electrode was imported into commercial software package Avizo V9.4 (Avizo, Thermo Fisher Scientific, Waltham, Massachusetts, USA) for microstructural characterisation and tortuosity factor measurement, which can also be achieved by using open-source software TauFactor^45^. A detailed thresholding segmentation workflow and the validation are illustrated shown in Supplementary Fig. 1. The pore size distribution was analysed using the Local Thickness plug-in from the open-source software Fiji^46^. The shape and orientation of NMC particles were extracted by calculating the moments of inertia of its ellipsoid template and the eigenvalues of the covariance matrix^47^. The sphericity of the NMC particles is presented in terms of the ratio between the minor and major axes of their best-fit ellipsoid. The graded-microstructure electrodes were artificially obtained by morphological operation.

### Morphological operation for graded-structures

The graded-structures were obtained by voxel manipulation based on the as-prepared 3D microstructure. Specifically, dilation and erosion were operated to enlarge and shrink the target phase by pushing or pulling the voxels at the phase boundary using a structure element (SE). The algorithms of these morphological operations can be found here^37,38^. To obtain a graded-particle volume, the original particles were firstly eroded by approx. 2 μm. Small particles (2−3 μm), which were directly taken from the as-prepared data, were spatially transformed (rotation, flip, etc.) and added into the non-particle region so as to maintain the same volume fraction of the active material, which can be quantified by image analysis tools in Avizo. For graded-porosity, all the boundary voxels of the pore phase in the respective modified region were pushed outward by 250 nm using dilation operation, while the voxels constituting the active materials were “locked” so that they cannot be affected, which means the mass loading of the electrode remains unchanged, and it is just the relative movement of the pore/CBD boundaries.

### 3D physics-based microstructure-resolved model

The reconstructed 3D volume of the NMC111 electrode (43 μm × 43 μm × 50 μm) was first segmented into three phases (i.e. active material, pore and CBD) based on greyscale thresholding (details about the thresholding workflow is shown in Fig. 3 and Supplementary Note 1), and was then imported into the commercial software package Simpleware ScanIP N-2018.03 for meshing. The physical size of the mesh element was adjusted according to the feature size of each phase and conformal nodes were ensured at the boundaries. The meshed volume consisted of 30 million tetrahedral elements and is shown in Supplementary Fig. 3.

The electrochemical model of the lithium metal half-cell was mathematically developed via a series of partial differential equations (PDEs) based on the generalised Poisson−Nernst−Planck (gPNP) equations^48^, which is a derivative of the Newman model^49^, implemented using Mathematics/General Form PDE module in the commercial software package COMSOL Multiphysics V5.3. Balance equations for the species Li^+^, PF_6_^−^, e^−^ and Li were solved in the entire simulation domain. Concentrated solution theory and electro-neutrality were implemented to describe mass transport in the electrolyte^48,50^, while Fick’s law was used for Li diffusion in the active material particles and Ohm’s law for electron transport in the CBD phase and active material particles. The charge-transfer reaction follows a kinetic expression originating from non-equilibrium thermodynamics, resulting in a Butler−Volmer-type expression. Details of the physical equations, boundary conditions and input parameters are shown in Supplementary Fig. 4 and Supplementary Tables 1−3. It is noted that no volume-averaged parameters (e.g. porosity and effective transport parameters) are used in the microstructure-resolved model.

## Supplementary information

Supplementary Information

Peer Review

## Data Availability

All data presented in this manuscript are available from the corresponding author upon request.

## Code availability

The code for the 3D electrochemical modelling is available from the corresponding author upon request.
