# Supplementary material for: 3D microstructure design of lithium-ion battery electrodes assisted by X-ray nano-computed tomography and modelling
Source: Nat Commun. 2020 Apr 29;11:2079. doi: 10.1038/s41467-020-15811-x (PMC7190643; doi:10.1038/s41467-020-15811-x)
Supplement: Supplementary file 1 — Supplementary Information [file 41467_2020_15811_MOESM1_ESM.pdf]

Supplementary information

3D microstructure design of lithium-ion battery electrodes assisted by X-ray nano-computed tomography and modelling

Lu et al.

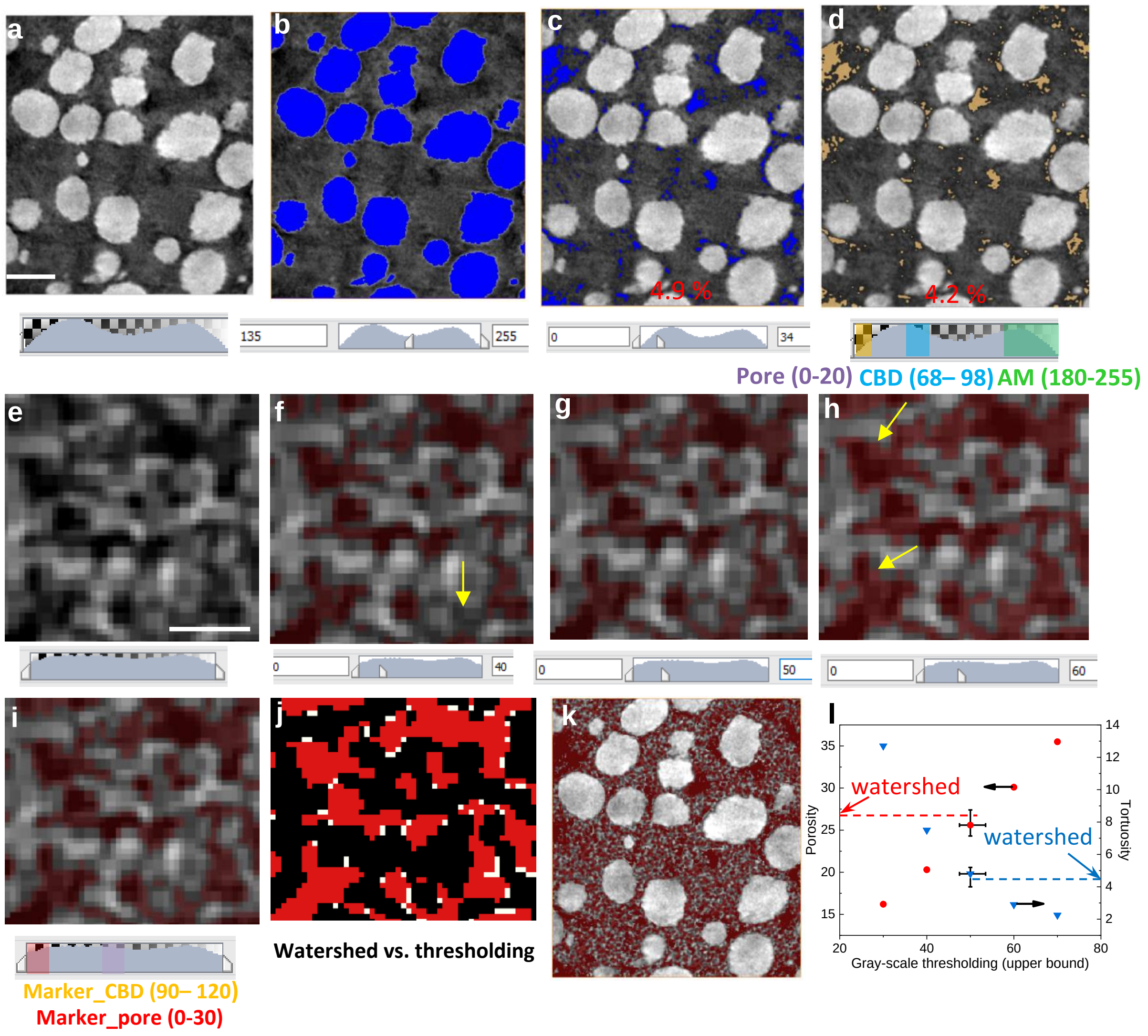

Supplementary Figure 1. Three-phase segmentation workflow using thresholding and the comparison with the marker-based watershed segmentation method. **a** The raw data of the reconstructed gray-scale image; **b** thresholding segmentation of the active material; **c** thresholding segmentation of the macro-pore phase; **d** macro-pore segmented by marker-based watershed method, with the histogram showing the marker range of each phase; **e** magnified image of the CBD phase; **f**, **g**, **h** micro-pore phase segmentation using low, medium and high thresholding range. The yellow arrows point out the under/over segmented region respectively; **i** resultant micro-pore segmented by marker-based watershed method. **j** The difference between thresholding and watershed segmentation is highlighted by white pixels; **k** overview of the pore phase segmented by thresholding method; l the resultant porosity and tortuosity of the pore phase segmented by different thresholding range, along with the uncertainty range in this study. Scale bar represents 7 µm in **a** and 2 µm in **e**.

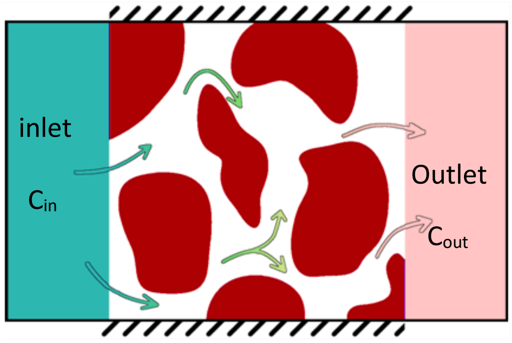

Supplementary Figure 2. Schematic figure showing the diffusion driven by concentration gradient in the porous electrode.

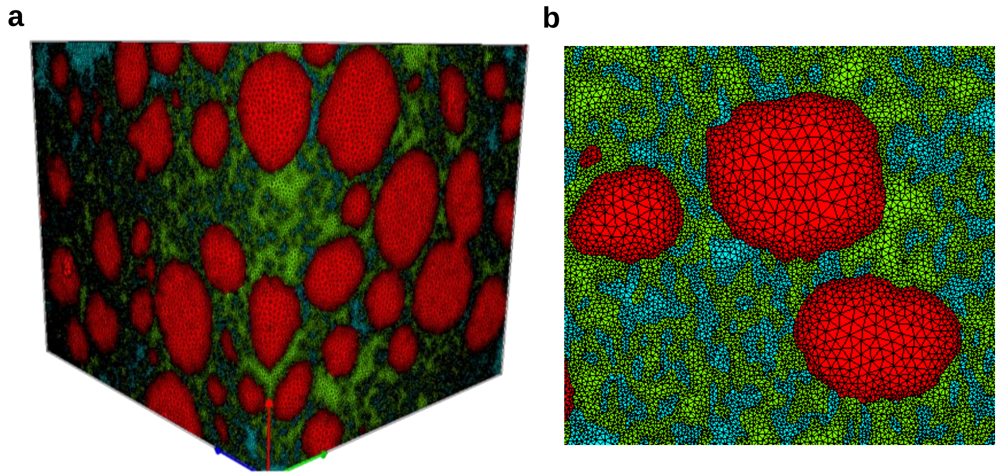

Supplementary Figure 3. The segmented volume of the electrode is meshed into three phases (red: active material; green: CBD; cyan: pore). Panel **b** is a magnified image of the meshed volume in **a** showing the physical size of the mesh element, which is adjusted according to the feature size of different phases.

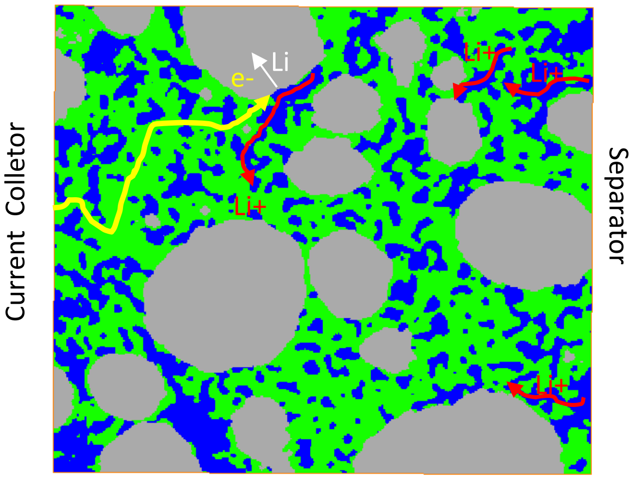

Supplementary Figure 4. Schematic of the physical process and the solution domain of the Li-ion battery model, in which the colours stand for grey: active material (Li intercalation and electrons transport); blue: pore (Li^+ transport); green: CBD (electrons transport).

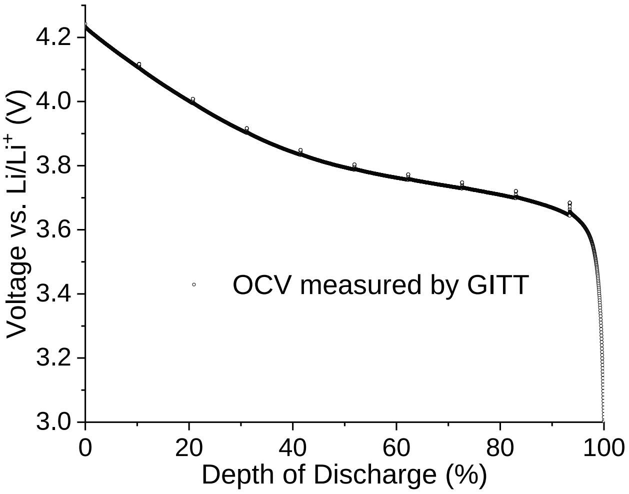

Supplementary Figure 5. Open-circuit voltage measured by GITT used for the modelling.

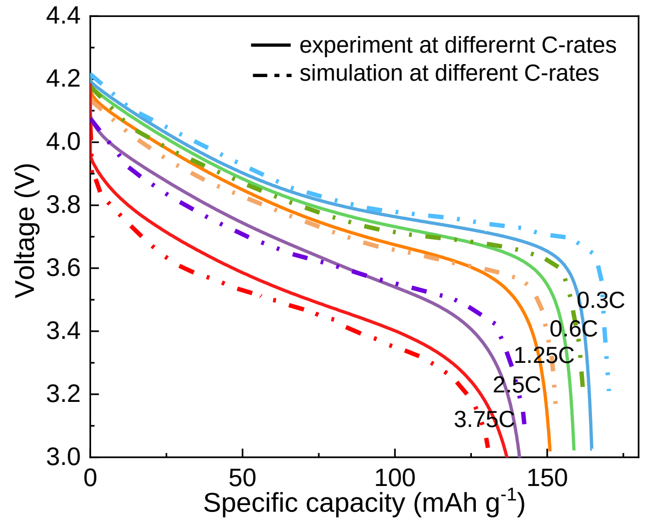

Supplementary Figure 6. Comparison of the experimental (solid) and simulated (dashed) discharge performance of the as-fabricated NMC111 cathode at various C-rates.

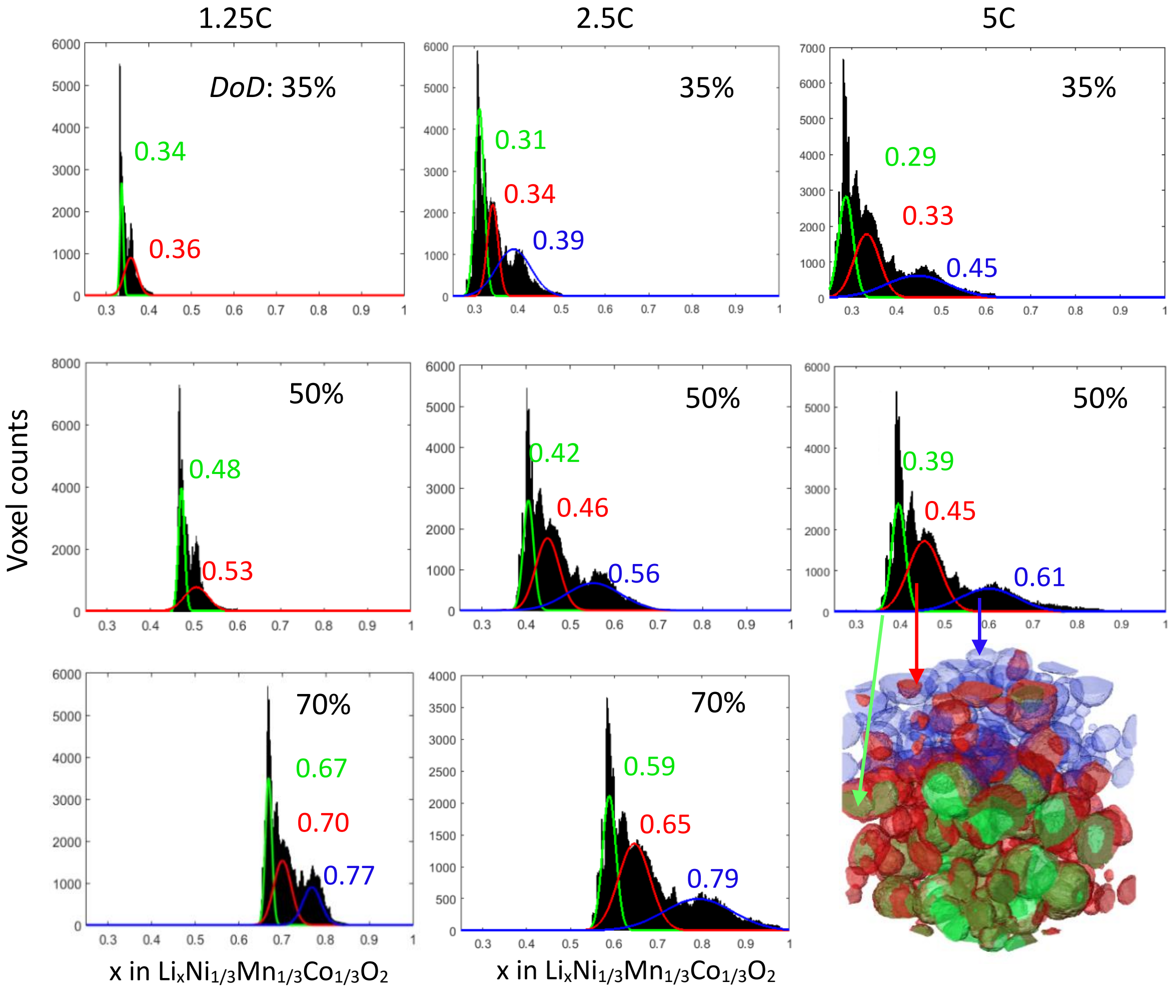

Supplementary Figure 7. Histogram of the state-of-lithiation (*SoL*) at depth-of-discharges (*DoD*s) of different discharging rates in the active material.

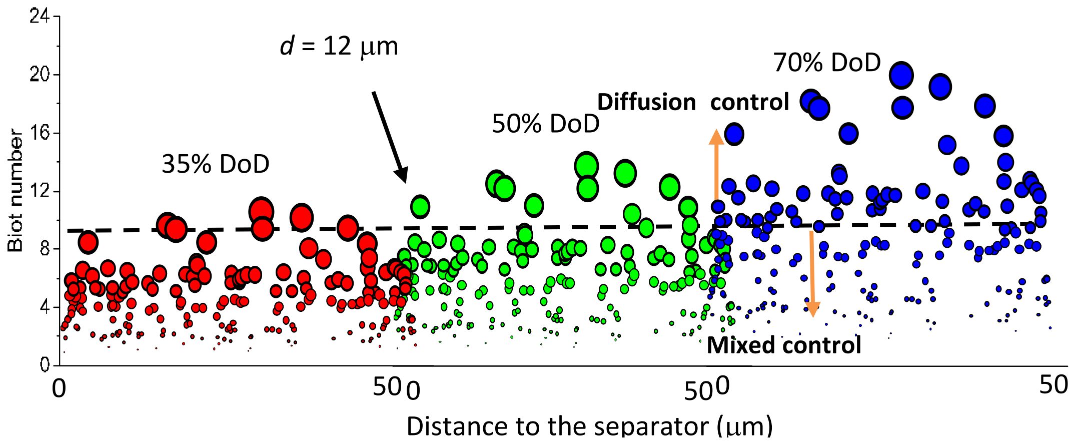

Supplementary Figure 8. Plot of the Biot number across different *DoDs*, inferring the relative rates of solid-state diffusion and interfacial reaction linked with the particle size.

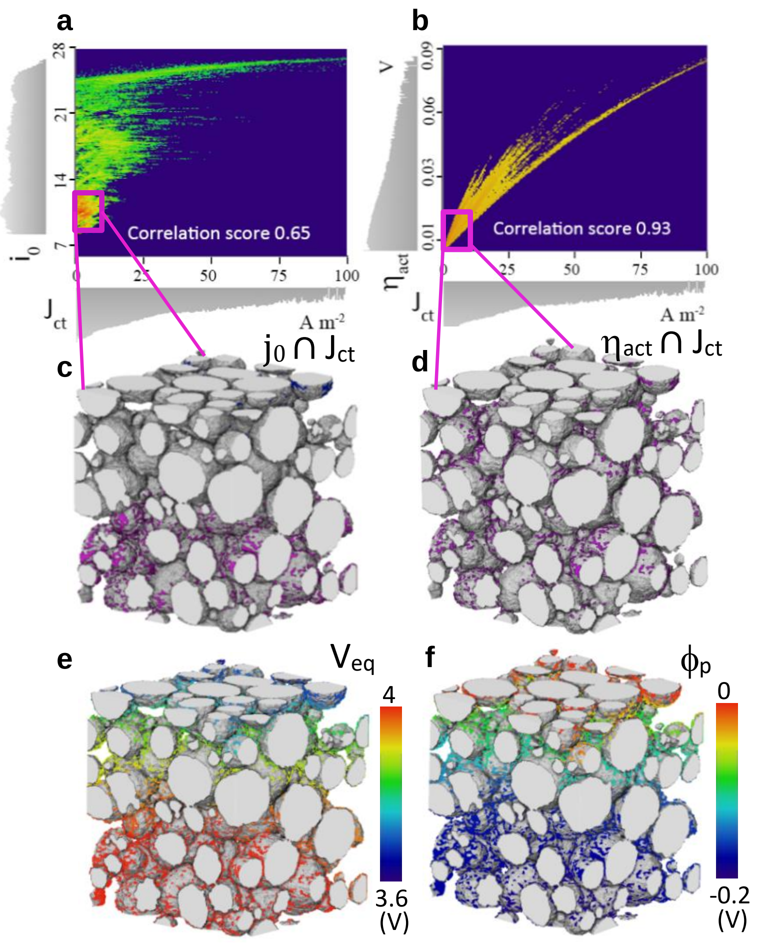

Supplementary Figure 9. 2D histogram correlation to find the dependence of charge transfer current density J_{ct} on exchange current density j_0 and activation overpotential η_{act} respectively. **a** Voxel correlation plot for J_{ct} and j_0 with highly correlated region shown in **c**; **b** voxel correlation plot for J_{ct} and η_{act} with highly correlated region shown in **d**; spatial distribution of equilibrium potential V_{eq} and potential in the interfacial electrolyte ϕ_p are shown in **e** and **f** respectively.

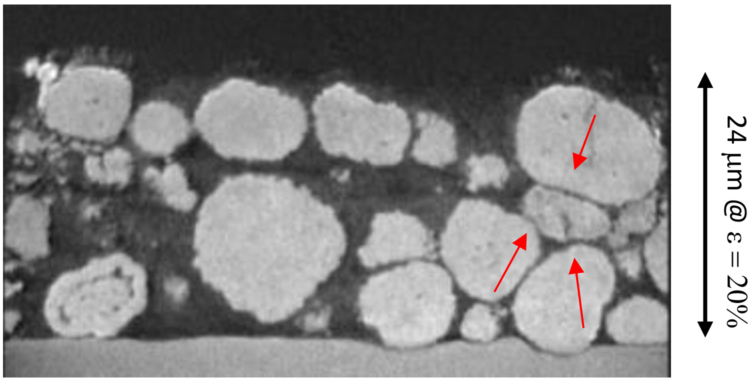

Supplementary Figure 10. X-ray tomographic slice of the 1 mAh cm^{-2} electrode after calendering at ε = 20%. Red arrows point out the 'crushed' CBD and the decimation of porosity, resulting in loss of electrochemical-active reaction sites and exacerbated structural heterogeneity.

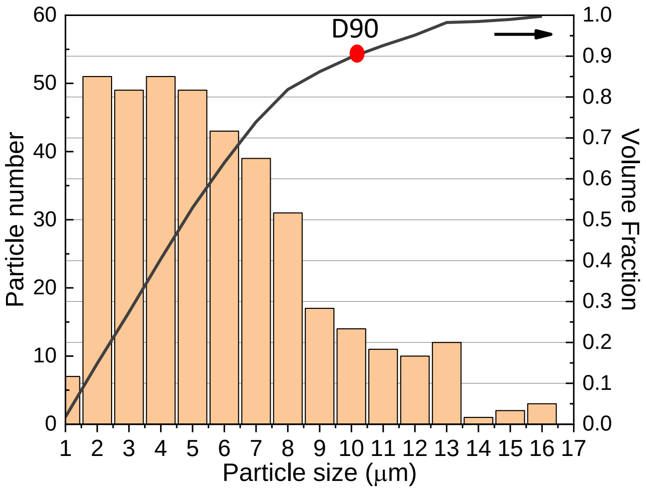

Supplementary Figure 11. Particle size distribution of the electrodes in Fig. 9l.

Supplementary Tables

Supplementary Table 1. Set of model equations, organised for each subdomain and reporting the associated dependent variable, balance equation, flux expression and additional relationships among field variables.

Pore (Electrolyte)		
Species	Balance equation	Flux expression
Li^+ ($\tilde{\mu}_p^*$)	$F\frac{\partial c}{\partial t}+\nabla\cdot J_p=0$ (1)	$J_p=-t_p\sigma_{io}\nabla\tilde{\mu}_p^*$ (2)
X^- (c)	$-F\frac{\partial c}{\partial t}+\nabla\cdot J_n=0$ (3)	$J_n=\frac{1}{t_p}F\tilde{D}\nabla c-(1-t_p)\sigma_{io}\nabla\tilde{\mu}_p^*$ (4)

Solid particles		
Species	Balance equation	Flux expression
Li (c_s)	$F\frac{\partial c_s}{\partial t}+\nabla\cdot J_s=0$ (5)	$J_s=-F\widetilde{D}_s\nabla c_s$ (6)
e^- ($\widetilde{\mu}_e^*$)	$\nabla\cdot J_e=0$ (7)	$J_e=-\sigma_e\nabla\widetilde{\mu}_e^*$ (8)
CBD		
Species	Balance equation	Flux expression
e^- ($\widetilde{\mu}_e^*$)	$\nabla\cdot J_e=0$ (9)	$J_e=-\sigma_e\nabla\widetilde{\mu}_e^*$ (10)
Charge-transfer kinetics at electrolyte/particles interface		
Kinetics	$J_{ct}=j_0\left[exp(\frac{\alpha F}{RT}(\tilde{\mu}_p^*-\tilde{\mu}_e^*+V_{ref}))-exp(-\frac{(1-\alpha)F}{RT}(\tilde{\mu}_p^*-\tilde{\mu}_e^*+V_{ref}))\right]$ (11)<br>$j_0=j_{00}c^{\alpha}c_s^{\alpha}(c_s^{\max}-c_s)^{1-\alpha}$ (12)	

Supplementary Table 2. Set of boundary conditions, where *n* is a unit vector pointing: i) from the separator to the electrode domain, ii) from the electrolyte phase to the active material particles, iii) from the electrode to the current collector. No flux conditions are applied elsewhere.

(i)Separator/electrode boundary	$\tilde{\mu}_p^*=0$ (reference potential), $n\cdot J_n=0$, $n\cdot J_s=0$, $n\cdot J_e=0$
(ii)Electrode (electrolyte/particles interface)	$n\cdot J_p=J_{ct}$, $n\cdot J_n=0$, $n\cdot J_s=J_{ct}$, $n\cdot J_e=J_{ct}$
(iii)Electrode/current collector boundary	$n\cdot J_p=0$, $n\cdot J_n=0$, $n\cdot J_s=0$, $n\cdot J_e=\frac{I}{A_e}$<br>$I=(c_s^{acc}-c_s^{in})v_{AM}V\frac{F}{3600}\frac{C_{rate}}{A}$ (13)<br>$c_s^{acc}=c_s^{\max}\times SOL_{acc}$, $c_s^{in}=c_s^{\max}\times SOL_{in}$

Supplementary Table 3. Simulation parameters

Parameters and domains	
Active Material	
D_s/m^{2}s^{-1}	Cui et al.[1]
σ_e/S m^{-1}	1.039×10^{-3} [2]
c_s^{max}/mol m^{-3}	24792
SOL_{in}	0.2
SOL_{max}	1
Electrolyte	
$\widetilde{D}$/m^{2}s^{-1}	Supplementary Equation 18 [3]
σ_{io}/S m^{-1}	Supplementary Equation 19 [3]
c_{ey}/mol m^{-3}	1000 [4]
t_p	0.38

CBD	
σ_e/S m^{-1}	0.375×10^{3} [5]
Reaction kinetics	
j_{00}/A m$^{2.5}$mol$^{-1.5}$	5.06×10^{-6} [4]
α	0.5
F/C mol^{-1}	96485
R/J mol^{-1} K^{-1}	8.314
T/K	298

Supplementary Table 4. Nomenclature

variable/parameters	
OCV	open circuit voltage, V
J_p	flux of positive ion (Li^+), A m^{-2}
J_n	flux of negative ion (X^-), A m^{-2}
J_e	flux of electrons (e^-), A m^{-2}
J_s	flux of lithium ion intercalated in the active material, A m^{-2}
$\tilde{\mu}_p^*$	reduced electrochemical potential of positive ions in the electrolyte, V
$\tilde{\mu}_e^*$	reduced electrochemical potential of electrons in their conducting phase, V
c	concentration of the electrolyte salt, mol m^{-3}
c_{ey}	initial electrolyte concentration, mol m^{-3}
c_s	lithium concentration in the active material, mol m^{-3}
c_s^{max}	maximum lithium concentration in the active material, mol m^{-3}
c_s^{in}	initial lithium concentration in the active material, mol m^{-3}
c_s^{acc}	accessible lithium concentration in the active material, mol m^{-3}
SOL_{in}	initial state of lithiation in the active material
SOL_{acc}	accessible state of lithiation in the active material
t_p	transference number of positive ion
σ_{io}	ionic conductivity in the electrolyte, S m^{-1}
$\widetilde{D}$	ambipolar diffusion coefficient of the binary electrolyte, m^{2} s^{-1}
σ_e	electronic conductivity of the solid material, S m^{-1}
D_s	solid state diffusion coefficient in active material, m^{2} s^{-1}
j_{00}	reaction rate constant, A m$^{2.5}$mol$^{-1.5}$
j_0	exchange current density, A m^{-2}
J_{ct}	charge transfer density at the reacting area, A m^{-2}
α	Transfer coefficient for intercalation half-reaction
I	applied current, A
A_e	area of the electron conducting materials on the inlet surface
v_{AM}	volume fraction of the active material in the electrode
V	total volume of the electrode, m^{3}
A	cross sectional area of the inlet surface, m^{2}
F	Faraday constant, C mol^{-1}
T	temperature, K
R	gas constant, J mol^{-1} K^{-1}
V_{ref}	equilibrium potential at the active material/electrolyte interface, V

Supplementary Note 1. Justification of image segmentation techniques

The gray-scale reconstructed data have clearly-defined boundaries between active materials and the other phases, which can be visualised in Supplementary Figure 1a and supported by the separated peaks in the histogram below it. Thus, the active material (bright phase) was firstly segmented using the thresholding range from the mid-point of two peaks (valley) to the high end to cover the second peak (Supplementary Figure 1b). However, the pore and CBD phases cannot be separated from the histogram, represented by the first peak on the left. Two reasons explain this: 1) the resolution is not high enough to resolve the nano-scale pores which are smeared with CBD; 2) even the macro-pores are darker in the data, but they cannot be reliably identified on the histogram due to the low volume fraction. However, at this step, it is still possible to segment the macro-pore from the remaining mixed phase due to its distinct contrast. An ideally-segmented macro-pore using the threshold value (0-34) (Supplementary Figure 1c) was compared with the marker-based watershed segmentation method [1] (Supplementary Figure 1d). This technique segments the data by growing from initially defined seeds in each region (the grayscale range defining the seeds in each phase is shown by the coloured overlay on the histogram), guided by the gradient of the gray-scale image, thus reliable boundaries between each phase can be obtained. It is found that the macro-pores segmented using two methods are very similar, visually judged by the segmented data and volume fraction (4.9% vs 4.2%).

The second step is segmenting the nano-pores after superimposing the stand-alone CBD scan with the original electrode. A higher magnification image of the CBD phase and the histogram after superimposition is shown in Supplementary Figure 1e. It is noted that the histogram is flattened, but this does not affect segmentation of the active material particles from the remaining phases, which is inherited from step one. In this study we chose “0-50” as the thresholding range to separate nano-pores from the CBD (shown in Supplementary Figure 1g), in comparison to the cases of under-segmentation (0-40, 20% narrower, Supplementary Figure 1f) and over-segmentation (0-60, 20% wider, Supplementary Figure 1h) of this phase (pointed out by yellow arrows in each subset). The marker-based watershed is also added into comparison (Supplementary Figure 1i). Supplementary Figure 1j highlights the segmentation difference (white voxels) between the thresholding (as-used, 0-50, Supplementary Figure 1g) and the watershed method; an insignificant disparity is found. Supplementary Figure 1k displays the overview of the segmented pores in the data. Supplementary Figure 1l compares the porosity and tortuosity factor obtained by different thresholding range. It is found that the resultant porosity is linearly dependent on the thresholding range. The x-axis error bar represents the uncertainty range when choosing the thresholding range by the authors, resulting in a + 1.8% to -1.3% variation on porosity and + 0.4 to -0.8 variation on tortuosity factor, statistically insignificant as a consequence of the good quality and contrast of the data. In addition, the porosity and tortuosity obtained by marker-based watershed segmentation are shown as red and blue dashed lines, close to what

we obtained using the thresholding method. Thus, it is reliable to use this thresholding method to separate the three phases using the workflow discussed above on this data.

Supplementary Note 2. Li^+ ion diffusion and tortuosity factor measurement using continuum CFD simulation

The Li^+ ion flow rate driven by a concentration gradient in a fully porous volume is described by Fick's law as

$$Q_e = AD\nabla c \tag{14}$$

where *D* is the diffusion coefficient, ∇c is the concentration gradient and *A* is the cross section area. For Li^+ ion flow in a porous medium, Supplementary Equation 14 is modified as

$$Q_p = \frac{\varepsilon}{\tau_c} AD\nabla c \tag{15}$$

where ε is the porosity, τ_c is the diffusion-based tortuosity factor that can be measured by dividing Q_p by Q_e, giving the effective transport parameter thereby the tortuosity factor,

$$\frac{Q_p}{Q_e} = \frac{\varepsilon}{\tau_c} \tag{16}$$

Supplementary Note 3. The electrochemical model of Li-ion battery simulation

The model describes the conservation of species in a Li-ion battery electrode made of a liquid electrolyte, which contains a Li^+/X^- salt, solid particles, which store Li and allow the transfer of electrons e^-, and CBD, which provides a preferential pathway for e^- transport. The charge-transfer reaction taking place at the particle/electrolyte interface is:

$$Li^+_{(ey)} + e^-_{(cbd+s)} \leftrightarrow Li_{(s)} \tag{17}$$

where *ey*, *s* and *cbd* stand for the electrolyte, solid and CBD phase. The 2D schematic below presents the solution domain and the basic physics of Li-ion battery modelling (half-cell).

The electrochemical model of the Li-ion battery (half-cell) was mathematically developed via a series of partial differential equations (PDEs) based on the generalised Poisson-Nernst-Planck (gPNP) equations[6], which is a derivative of the Newman's model[7]. Balance equations for the species Li^+, PF_6^-, e^- and Li were solved in the entire simulation domain. Concentrated solutions and electro-neutrality were implemented to describe the mass transport in the electrolyte while the Fick law was used for Li in the solid particles and Ohm law was used for e^- transport in the CBD phase as well as solid particles. The charge-transfer reaction follows a

kinetic expression coming from non-equilibrium thermodynamics, resulting in a Butler-Volmer-like expression. The details of the mathematics, boundary conditions and input parameters are listed in Supplementary Table 1, 2 and 3 respectively. The nomenclature is reported afterwards.

It is noted that all the fluxes are expressed as (positive) current densities, thus simplifying the coupling with the charge-transfer reaction at the electrolyte/particles interface. Similarly, the balance equations are reported in such a current basis. The initial conditions were set as follows:

$$c = c_{ey}\,;\ \tilde{\mu}_p^* = 0\,;\ c_s = c_s^{in}\,;\ \tilde{\mu}_e^* = OCV$$

The OCV vs. DoD was measured using Galvanostatic intermittent titration technique (GITT) method on the coin cells assembled (Supplementary Figure 5) and used to compute the reference potential V_{ref} as a function of the local state of lithiation *SOL*.

The ambipolar diffusion coefficient of the binary electrolyte was defined as a function of the electrolyte concentration (*c*) and temperature (*T*) as[3]:

$$\tilde{D} = 10^{-4} \times 10^{-4.43-(54/(T-229-5.0\times10^{-3}c))} - 0.22\times10^{-3}c \tag{18}$$

The ionic conductivity for the binary electrolyte was defined as a function of the electrolyte concentration (*c*) and temperature (*T*) as[3,8]:

$$\begin{aligned}\sigma_{io} = 10^{-4}\times c(&-10.5+0.668\times10^{-3}c+0.494\times10^{-6}c^{-2}\\ &+0.074T-1.78\times10^{-5}cT-8.86\times10^{-10}c^2T\\ &-6.96\times10^{-5}T^2+2.8\times10^{-8}cT^2)^2\end{aligned} \tag{19}$$

The NMC solid state diffusivity (D_s) as a function of the lithiation state used in the simulation was experimentally measured by GITT method[1].

The full list of the simulation parameters is presented in Supplementary Table 3. It is noted that no volume-averaged parameters (e.g. porosity and effective parameters) were used in the microstructure-resolved model.

Good agreement is observed between the predicted performance and the experimental data. This highlights the advantage of image-based modelling as no volume-averaged parameters were used. At higher C-rate (3.75C), the simulated result slightly deviates from the experiment, which is speculated to arise from the accumulated heat effect caused by the current flow. However, given that no experimental fitting and parameter refinement is employed, this 3D microstructure-resolved model provides unique capability for electrode design and optimisation with a maximum of 7 % error in the predicted specific energy and power density compared to experiment.

Supplementary Note 4. Electrochemical Biot number

The intra- and inter-particle heterogeneity in *SoL* is mainly attributed to the relative interplay between solid-state diffusion in the particles and the charge transfer reaction at the electrolyte/electrode interface, which can be evaluated by the electrochemical Biot number (B)[9,10] for the fabricated NMC particles (Supplementary Figure 8). It is found that at 35% *DoD*, the majority of the NMC particles are under mixed control of the surface reaction kinetics and solid-state diffusion; as the discharge reaches 50% *DoD*, the *SoL* of the particles larger than 12 μm is deleteriously affected due to the relatively sluggish solid-state diffusion; at 70% *DoD*, solid-state diffusion resistance dominates half of the total NMC particles for the as-prepared electrode. This explains why the discrepancy of *SoL* between small and larger particles becomes more severe with the discharge time.

Supplementary Note 5. 2D histogram correlation to understand the charge transfer heterogeneity

2D histogram correlation: two scalar fields are spatially correlated on a voxel-by-voxel basis; the scalar values of the paired-voxel in volume one and volume two determines the coordinate in the resultant 2D histogram, and the colour indicates the total number of identical voxel-pairs. This is plotted for relationships of j_0 vs. J_{ct} and η_{act} vs. J_{ct} in Supplementary Figure 9a and b respectively. It is observed that J_{ct} has a much higher overall correlation score with η_{act} than with j_0 (0.93 *vs.* 0.65); in other words, the charge transfer current density (J_{ct}) is more strongly correlated with the activation over potential η_{act} than the exchange current density j_0. Two regions on the 2D correlated histograms are selected and the corresponding voxels are visualised in Supplementary Figure 9c and d. It is noticed that the highly correlated region between j_0 and J_{ct} mainly resides in the bottom half of the electrode thickness (closer to the current collector), where the rate of electrochemical reaction is lower due to depletion of reactants. In contrast, the correlation score is generally above 0.9 across the η_{act} and J_{ct} histograms. The co-localised voxel pairs distribute uniformly from the top (separator) to the bottom (current collector). In order to further explore the heterogeneous distribution of η_{act}, the two primary electrochemical field variables linked to η_{act} are mapped spatially onto the solid particles: the equilibrium potential V_{eq} at the reaction interface (Supplementary Figure 9e) and the interfacial potential in the electrolyte ϕ_p (Supplementary Figure 9f). It is observed that generally they distribute monotonically along the through-thickness direction: V_{eq} is inversely related to the lithium concentration c_s at the particle surface which is highest close to the separator and ϕ_p generally varies according to Ohm's law. However, they have opposite contributions to the magnitude of η_{act}. The combination of these two electrochemical state variables leads to the heterogeneous distribution of η_{act} and thus J_{ct}.

Supplementary References

1 Cui, S. *et al.* Optimized Temperature Effect of Li-Ion Diffusion with Layer Distance in Li (NixMnyCoz) O2 Cathode Materials for High Performance Li-Ion Battery. *Adv. Energy Mater.* **6**, 1501309 (2016).

2 Park, M., Zhang, X., Chung, M., Less, G. B. & Sastry, A. M. A review of conduction phenomena in Li-ion batteries. *J. Power Sources* **195**, 7904-7929 (2010).

3 Cai, L. & White, R. E. Mathematical modeling of a lithium ion battery with thermal effects in COMSOL Inc. Multiphysics (MP) software. *J. Power Sources* **196**, 5985-5989 (2011).

4 Danner, T. *et al.* Thick electrodes for Li-ion batteries: A model based analysis. *J. Power Sources* **334**, 191-201 (2016).

5 Liu, G. *et al.* Effects of Various Conductive Additive and Polymeric Binder Contents on the Performance of a Lithium-Ion Composite Cathode. *J. Electrochem. Soc.* **155**, A887-A892 (2008).

6 Lai, W. & Ciucci, F. Mathematical modeling of porous battery electrodes—Revisit of Newman's model. *Electrochim. Acta* **56**, 4369-4377 (2011).

7 Doyle, M., Fuller, T. F. & Newman, J. Modeling of galvanostatic charge and discharge of the lithium/polymer/insertion cell. *J. Electrochem. Soc.* **140**, 1526-1533 (1993).

8 Ding, M. S. *et al.* Change of Conductivity with Salt Content, Solvent Composition, and Temperature for Electrolytes of LiPF6 in Ethylene Carbonate-Ethyl Methyl Carbonate. *J. Electrochem. Soc.* **148**, A1196-A1204 (2001).

9 Tsai, P.-C. *et al.* Single-particle measurements of electrochemical kinetics in NMC and NCA cathodes for Li-ion batteries. *Energy Environ. Sci.* **11**, 860-871 (2018).

10 He, F. *et al.* Determination of Electrochemical Kinetic Property for Mixed Ionic Electronic Conductors from Electrical Conductivity Relaxation Measurements. *J. Electrochem. Soc.* **162**, F951-F958 (2015).
