# Supplementary material for: 3D microstructure design of lithium-ion battery electrodes assisted by X-ray nano-computed tomography and modelling
Source: Nat Commun. 2020 Apr 29;11:2079. doi: 10.1038/s41467-020-15811-x (PMC7190643; doi:10.1038/s41467-020-15811-x)
Supplement: Supplementary file 2 — Peer Review [file 41467_2020_15811_MOESM2_ESM.pdf]

Reviewers' comments:

Reviewer #1 (Remarks to the Author):

First, let me congratulate the authors for doing a very comprehensive and thorough work on investigating transport and diffusion at both particle and electrode level by quantitatively resolving the micro (nano)structure of the composite electrode using X-ray CT. The results will be very useful to the lithium ion battery community in advancing the knowledge of electrode design specifically meeting the requirements such as fast charging, capacity utilization and addressing issues such as capacity degradation. and thermal safety. Now I will provide specific examples justifying my observation.

1. This is a paper that discusses all aspects and important parameters pertaining to heterogeneity in Li-ion transport in LIB electrodes, (i) Variation of State of lithiation (SoL), (ii) Charge transfer and current density distribution at the pores and interface at various C-rates, (iii) effect of graded electrode porosity and particle size distribution and finally (iv) the effect of calendering on the performance.

2. In addition, the manuscript provides practical suggestion on how to optimize the power and energy at high discharge rates by optimizing the microstructure such as gradient porosity.

3. Overall there is tremendous value on observing Li-ion transport across the through plane (thickness) in 3D and observing the variation of transport parameters near the separator till the current collector.

4. Regarding the technique although X-ray CT has been used extensively by others but dual scan superposition (DSS) methods brings much needed fidelity and resolution to resolve the microstructure for undertaking 4D X-ray CT.

Couple of points-
1. Fig. 2f and h- It's interesting that authors have discussion of the shape (aspect ratio) and sphericity distribution. This was for the pristine or uncycled electrode. Its possible that under long term cycling the majority of particles will deviate from spherical shape due to stress etc.. Similar effect has been reported from XANES tomography which has even higher spatial resolution ~ 30nm [Nano Lett. 2014, 14, 4334−4341]. This provides a good discussion theme for discussion for the changes in particle morphology with cycling which can effect the porosity, density etc..

2. Fig. 3- Charge transfer- Jct across the electrolyte-particle interface : this doesn't show much variation with C-rates unlike electrolyte concentration or Li-ion flux ?

Reviewer #2 (Remarks to the Author):

This manuscript reports a dual-scan superimposition technique to image the features of the NMC cathode material by X-ray tomography. Electrochemical model, built based on the features obtained from imaging, is employed to study how the micro pores, particle heterogeneity (size, distribution) and the calendaring affect the electrode’s performance (capacity, rate performance and/or energy density). However, many questions are remained as unclear in terms of data interpretation before it can be considered to be published.

1. In line 79, the authors introduce a dual-scan superimposition method. Details have been given in

the paragraph about the second scan. How about the first scan? What are the main differences between the first and second scans? Experimental parameters about the first and second scans are also missing in the Methods section, which should be added.

2. As far as the referee understand, Fig. 1a and 1b come from the results of X-ray micro-CT from a cylindrical battery, while Fig. 1e to 1g are from the results of cathode sheet prepared by doctor blade from X-ray nano-CT. It is misleading if these cathode sheets are not directly obtained from a cylindrical battery as the red lines show in Fig.1. In addition, the experimental parameters of the X-ray micro CT are missing in the Method section.

3. The authors mentioned that "conservation of species (i.e., Li+, PF6-, e- and Li) was enforced in the entire simulation domain". But several species are not constant during the electrochemistry. For instance, in the simulation domain Li+ ions are inserted in the NMC particles and electrons are provided from the other electrode and are consumed in this domain. How do the authors explain this?

4. Regarding the figures in this manuscript, 1) the region of R1 to R4 in Fig.7 is not clearly indicated in Fig. 7a-c; 2) In Fig. 8, the labels number 1-4 on the dashed lines might not be correct, as number 2 and 3 are labeled on one dashed line (pink).

5. In line 463, the authors state that "a video has been added…". Unfortunately, the referee did not see such a video in the manuscript tracking system.

6. Suggested minor things: 1) Reference 13 and 15 are the same. 2) The preferential pathway of electron shown in Fig. S3 is not correct as electrons prefer to migrate along the carbon matrix instead of passing through the NMC particles.

Reviewer #3 (Remarks to the Author):

The manuscript entitled "Advanced Design of Lithium-ion Battery Electrodes assisted by 4D X-ray Nano-computed Tomography and Modelling" should be accepted to Nature Communications with revisions.

The manuscript can be subdivided into four main sections. The first section introduces a novel dual-scan superposition process to discriminate the binder phase from pore space in a LiNi1/3Mn1/3Co1/3O2 cathode. The technique implements a second X-ray nano-CT scan. The resulting X-ray measured microstructures capture detailed binder features previously measured using scanning electron microscopy. The paper's second section incorporates these microstructures into 3D electrochemical models. Initial modeling results using the reconstructed data indicates the particle distribution and orientation. Additionally, the model predicts how Li-ion transport losses throughout the electrode cause inhomogeneous electrode utilization. The manuscript's third section models a modified electrode particle distributions to predict the influence of particle-grading. Finally, an experimental time-lapse X-ray nano-CT experiment is used to quantify the effects of electrode calendaring.

The following are major revisions that need to be addressed:
1. Lines 100 and 448-449: Three-body thresholding is difficult, and can be influenced by ambiguities in the images and subjective decisions. The paper would benefit from further discussion to explain and interpret the three-body thresholding method. If there are three obvious peaks in the gray-scale data that clearly identify the respective regions, then this can just be stated in the manuscript. Otherwise, the thresholding scheme needs to be further documented.
2. Line 210-211, 454-455 and 38 in Supplemental: The authors implement dilute solution theory to model the Li-ion transport. The Poisson-Nernst-Planck equations used in the present study result in

different solution-phase potential drops than those derived from concentrated solution theory [1]. Concentrated solution theory is commonly used in Li-ion battery modeling, usually following the approach developed by Newman and colleagues. The paper must correct the citation in line 38 of the Supplemental material, which incorrectly references the Doyle-Fuller-Newman model as implementing dilute solution theory. Additionally, the authors found that the local discrepancy in electrolyte potential and charge-transfer current density are closely related (Lines: 216-217). Supporting text is needed in the paragraph (Lines 190-223) that discusses how using concentrated solution theory may affect the reported modeling results.

The following are minor revisions that need to be addressed:
3. Line 3: The first author Xuekun Lu is associated with the Electrochemical Innovation Lab twice (i.e., there are two number ones above their name).
4. Line 111: What is the program/calculation used to extract the "volume-averaged tortuosity factor".
5. Figure 6 d-e, g-h, and j-l need to state the C-rate and at what time the data is taken.
6. Lines 250-252 and 256-257: Particle size reduction (or morphological dilation) is nontrivial in a three-phase media. Additional text is required that discusses how the particle size is reduced near the separator "while maintaining the same particle volume fraction". Additional text is also required to support what the authors mean by "morphological dilation." The authors state that they shrink the CBD domain. Does this create more solid/electrolyte active lithiation sites?
7. Line 286-287: The authors state: "decreasing the CBD volume while maintaining the mass loading constant." The meaning of this sentence is unclear. To keep the mass loading constant did the authors increase the NMC volume fraction in this region? Do the authors instead mean that the NMC loading is held constant, while the CBD volume fraction is reduced?
8. Figure 9k illustrates the porosity and tortuosity as a function of strain. How are these parameters calculated? Are they averaged over the entire domain or just the cut plane illustrated in Fig. 9d-f.
9. Line 437: The word "voxel" is oddly typeset.
10. References: There are sometimes odd spaces before and after "– ". For example, see Line 487.
11. Line 491 (References): There are dots where there should be dashes.
12. Figure S7: What is the x-axis?

General thoughts:
13. The final section in the manuscript (Lines 330-381) seems tacked-on as opposed to a continuation of the paper. This section could be removed. Alternatively, the section could be strengthened by providing clear context relative to the body of the paper..
14. The figures are not colorblind friendly. The same tone of red and green are commonly used in the same plot.

[1] A.M. Bizeray, D.A. Howey, and C.W. Monroe. Resolving a discrepancy in diffusion potentials with a case study for Li-ion batteries. J. Electrochem. Soc., 163 E223-E229 (2016).

Reviewer #4 (Remarks to the Author):

This paper deals with some suggestions to improve the electrode design of NMC LIBs. To achieve this, a combination of visual 3D tomography technique, electrical measurement of coin cells and a physical chemical model in COMSOL was applied. To analyse the full structure of the NMC electrode a superimposition technique of high attenuation and low attenuation regions were overlaid. The model was used to identify better performances of the electrode while changing the ratio between pores and binder and applying graded particle sizes near the separator. Also the influence of calendaring of the NMC electrode is studied.
The final results of the paper regarding the calendaring for different cell types (high power / high energy) and the graded particle approach are relevant and presented well. However, the structure of the paper is very confusing. Some subsections may help to follow the different analysis and small

connecting paragraphs between the sections would improve continuous comprehension and readability. The title is about 4D tomography, normally the fourth dimension in the literature is time, but here no time elapsed measurement is shown.

First Part: 3D characterisation
The author point out, that X-ray can be used to get insight of material structure. But there are some limits and a novel technique was used to overcome the limits.
• This novel technique is not explained in detail. If it is about using a short exposure time and a long exposure time and overlaying this images, it is not novel it is like HDR images. In addition, is this process before or after reconstruction? What is the meaning of high confidence in the context of this measurement?
• Why didn't you use synchrotron X-ray to achieve a high contrast and resolution tomography?
• [p2; line 38] How is it possible to remove the NMC particles within a sample? If this is a physical process how do you ensure that it is not changing the structure?
• How can you be sure that the reconstructed data has no artefacts, void zones or noise? (compare 180° rotation vs. 360° rotation)
• More information about the reconstruction process is needed

Second Part: Microstructure and performance correlation
[p.5; line 4] Coin cells were prepared to show the correlation between model and experiment.
• Information about the coin cells experiment is missing (amount of measured coin cells, at which operating temperature). In addition, it is not clear if the model has the same material parameters as the used coin cell materials.

Third Part: Design and optimisation of next-generation electrodes
Some subsections would improve the readability (procedure and steps, results and impacts and finally suggestions)
It is shown, that the shrinking of the CBD (or increasing the porosity) has a high impact of the electrical performance and the capacity.
• Hence the quality (noise) and threshold of the reconstructed data has even a big influence on the results. This is not discussed and leads to a not trustworthy model. (250nm shrinking = 4 voxels = within measuring error?)
• [p11. Line 29] This is the case in the most of BMSs

Fourth Part: Combining 4D X-ray nano-CT and electrochemical performance
This should be the main part related to the title.
• It is not explicit said, what is the fourth dimension? If its time, there are no experimental data or in-situ measurements.

Fifth Part: Conclusion
• [p.14 line 15] This is not a new but maybe a relevant insight.

Reviewers' comments:

Reviewer #1 (Remarks to the Author):

First, let me congratulate the authors for doing a very comprehensive and thorough work on investigating transport and diffusion at both particle and electrode level by quantitatively resolving the micro (nano)structure of the composite electrode using X-ray CT. The results will be very useful to the lithium ion battery community in advancing the knowledge of electrode design specifically meeting the requirements such as fast charging, capacity utilization and addressing issues such as capacity degradation. and thermal safety. Now I will provide specific examples justifying my observation.

1. This is a paper that discusses all aspects and important parameters pertaining to heterogeneity in Li-ion transport in LIB electrodes, (i) Variation of State of lithiation (SoL), (ii) Charge transfer and current density distribution at the pores and interface at various C-rates, (iii) effect of graded electrode porosity and particle size distribution and finally (iv) the effect of calendering on the performance.

2. In addition, the manuscript provides practical suggestion on how to optimize the power and energy at high discharge rates by optimizing the microstructure such as gradient porosity.

3. Overall there is tremendous value on observing Li-ion transport across the through plane (thickness) in 3D and observing the variation of transport parameters near the separator till the current collector.

4. Regarding the technique although X-ray CT has been used extensively by others but dual scan superposition (DSS) methods brings much needed fidelity and resolution to resolve the microstructure for undertaking 4D X-ray CT.

The authors are grateful to the Reviewer's positive comments about the highlights of this work, which are indeed the outcome of years of effort in tackling the technical challenges. We hope that this study can benefit a great number of researchers and provide new insights into the research relating to battery electrode design and manufacturing for improved performance and safety.

Couple of points-

1. Fig. 2f and h- It's interesting that authors have discussion of the shape (aspect ratio) and sphericity distribution. This was for the pristine or uncycled electrode. Its possible that under long term cycling the majority of particles will deviate from spherical shape due to stress etc.. Similar effect has been reported from XANES tomography which has even higher spatial resolution ~ 30nm [Nano Lett. 2014, 14, 4334−4341]. This provides a good discussion theme for discussion for the changes in particle morphology with cycling which can effect the porosity, density etc..

It is really a good point to open a discussion in this aspect. The authors have read the listed reference and found that the voltage window for the cycling was quite large (2.5 V to 4.9 V) compared to the normal cutoff voltage, which probably explains why the morphological change occurred after the 1st cycle. But the authors agree that morphological change could happen due to long-term cycling. Newly added discussion regarding this effect have been highlighted in context

below Fig. 1.

2. Fig. 3- Charge transfer- Jct across the electrolyte-particle interface : this doesn't show much variation with C-rates unlike electrolyte concentration or Li-ion flux ?

The authors think C-rates do have an influence on Jct: at low C-rates, the magnitude of Jct is more uniform across the whole thickness compared to those at high C-rates, in which the reaction heterogeneity becomes very serious, as evidenced by the large variation of the Jct magnitude both at the same depth and along the thickness direction. The build-up of Jct gradient is an inevitable consequence of mass transport limitation that switch-offs the reaction sites in the vicinity of the current collector. To make this clearer, the authors have added complementary sentences in context above Fig. 3.

Reviewer #2 (Remarks to the Author):

This manuscript reports a dual-scan superimposition technique to image the features of the NMC cathode material by X-ray tomography. Electrochemical model, built based on the features obtained from imaging, is employed to study how the micro pores, particle heterogeneity (size, distribution) and the calendaring affect the electrode’s performance (capacity, rate performance and/or energy density). However, many questions are remained as unclear in terms of data interpretation before it can be considered to be published.

1. In line 79, the authors introduce a dual-scan superimposition method. Details have been given in the paragraph about the second scan. How about the first scan? What are the main differences between the first and second scans? Experimental parameters about the first and second scans are also missing in the Methods section, which should be added.

The scanning parameters were introduced in the *Methods* section (starting from the 4th sentence in the third paragraph). The energy, projection numbers were exactly the same between the two scans, while the first scan had a voxel size of 126 nm whereas it was 63 nm for the second scan (imaging CBD). Accordingly the exposure time for the CBD scan was longer than the electrode scan (40s vs 16s). The authors have added further detailed scanning information in the *Methods* as suggested by the Reviewer, and also in the second paragraph of the *Results* section to point to the *Method* section for detailed scanning information.

2. As far as the referee understand, Fig. 1a and 1b come from the results of X-ray micro-CT from a cylindrical battery, while Fig. 1e to 1g are from the results of cathode sheet prepared by doctor blade from X-ray nano-CT. It is misleading if these cathode sheets are not directly obtained from a cylindrical battery as the red lines show in Fig.1. In addition, the experimental parameters of the X-ray micro CT are missing in the Method section.

The main purpose of Fig. 1 is to conceptually illustrate the hierarchical length scale of the battery electrode, which is especially necessary for a comprehensive Journal like Nature Comm. As the Reviewer pointed out, the nano-CT data is not directly from cylindrical cell, but the 3D microstructure of the electrode, either from cylindrical cell or made by doctor blade, are similar in terms of the particle morphology and three phase arrangements, according to our previous scans. To

avoid confusion, a further clarification regarding this point "X-ray nano-CT was then conducted to capture the 3D microstructural details of the layered cell separately prepared by the doctor blade coating method (Fig. 1c)" has been added and highlighted in the first paragraph of *Results* section.

The scanning parameters of the micro-CT for the cylindrical cell have been added in the caption of Fig.1.

3. The authors mentioned that "conservation of species (i.e., Li+, PF6-, e- and Li) was enforced in the entire simulation domain". But several species are not constant during the electrochemistry. For instance, in the simulation domain Li+ ions are inserted in the NMC particles and electrons are provided from the other electrode and are consumed in this domain. How do the authors explain this?

The "conservation of species" here does not mean to say the species are conserved between anode and cathode, instead, it expresses that the consumption/production rate of each species at the reaction interface must be equal to (i.e. balance) the charge transfer current density (source term) while for the elements not adjacent to the reaction interface, the source term is zero. Thus, it is the conservation of each species with the source term from current density. To avoid confusion, the authors have rephrased the sentence as "Balance equations for the species Li^+, PF_6^-, e^- and Li were solved in the entire simulation domain."

4. Regarding the figures in this manuscript, 1) the region of R1 to R4 in Fig.7 is not clearly indicated in Fig. 7a-c; 2) In Fig. 8, the labels number 1-4 on the dashed lines might not be correct, as number 2 and 3 are labeled on one dashed line (pink).

Following the Reviewer's suggestion, the volumes in Fig.7a-c have now been labelled as R1, R2 and R3; In Fig. 8, the labels position are correct as number 2 and 3 are representing the same type of sample (high porosity) but at different C-rates. This was explained in the paragraph below Fig. 8.

5. In line 463, the authors state that "a video has been added…". Unfortunately, the referee did not see such a video in the manuscript tracking system.

Apologies, this line should have been deleted, and was left over from a previous iteration of the manuscript. This has now been revised.

6. Suggested minor things: 1) Reference 13 and 15 are the same. 2) The preferential pathway of electron shown in Fig. S3 is not correct as electrons prefer to migrate along the carbon matrix instead of passing through the NMC particles.

1) The authors thank the Reviewer for pointing out the repeated reference, which has been corrected. 2) The Fig. S3 (now Fig. S4) has been corrected thanks to the Reviewer's comment.

Reviewer #3 (Remarks to the Author):

The manuscript entitled "Advanced Design of Lithium-ion Battery Electrodes assisted by 4D X-ray Nano-computed Tomography and Modelling" should be accepted to Nature Communications with revisions.

The manuscript can be subdivided into four main sections. The first section introduces a novel dual-scan superposition process to discriminate the binder phase from pore space in a LiNi1/3Mn1/3Co1/3O2 cathode. The technique implements a second X-ray nano-CT scan. The resulting X-ray measured microstructures capture detailed binder features previously measured using scanning electron microscopy. The paper's second section incorporates these microstructures into 3D electrochemical models. Initial modeling results using the reconstructed data indicates the particle distribution and orientation. Additionally, the model predicts how Li-ion transport losses throughout the electrode cause inhomogeneous electrode utilization. The manuscript's third section models a modified electrode particle distributions to predict the influence of particle-grading. Finally, an experimental time-lapse X-ray nano-CT experiment is used to quantify the effects of electrode calendaring.

The following are major revisions that need to be addressed:
1. Lines 100 and 448-449: Three-body thresholding is difficult, and can be influenced by ambiguities in the images and subjective decisions. The paper would benefit from further discussion to explain and interpret the three-body thresholding method. If there are three obvious peaks in the gray-scale data that clearly identify the respective regions, then this can just be stated in the manuscript. Otherwise, the thresholding scheme needs to be further documented.

The authors agree with the Reviewer's comment about the segmentation method, which often raises concern about the analysis. Hence, following the Reviewer's suggestion, the authors have added a very detailed explanation and justification of the thresholding method used in this study (shown in Fig. S1 in SI). The text related to it is copied below, some additions are also added in the second paragraph in *Results* section, and also in *Methods* section, pointing to the SI for the readers who are interested.

"The gray-scale reconstructed data have clearly-defined boundaries between active materials and the other phases, which can be visualised in Fig. S1a and supported by the separated peaks in the histogram below it. Thus, the active material (bright phase) was firstly segmented using the thresholding range from the mid-point of two peaks (valley) to the high end to cover the second peak (Fig. S1b). However, the pore and CBD phases cannot be separated from the histogram, represented by the first peak on the left. Two reasons explain this: 1) the resolution is not high enough to resolve the nano-scale pores which are smeared with CBD; 2) even the macro-pores are darker in the data, but they cannot be reliably identified on the histogram due to the low volume fraction. However, at this step, it is still possible to segment the macro-pore from the remaining mixed phase due to its distinct contrast. An ideally-segmented macro-pore using the threshold value (0-34) (Fig. S1c) was compared with the marker-based watershed segmentation method [1] (Fig. S1d). This technique segments the data by growing from initially defined seeds in each region (the grayscale range defining the seeds in each phase is shown by the coloured overlay on the histogram), guided by the gradient of the gray-scale image, thus reliable boundaries between each phase can be obtained. It is found that the macro-pores segmented using two methods are very similar, visually judged by the segmented data and volume fraction (4.9% vs 4.2%).

The second step is segmenting the nano-pores after superimposing the stand-alone CBD scan with the original electrode. A higher magnification image of the CBD phase and the histogram after superimposition is shown in Fig. S1e. It is noted that the histogram is flattened, but this does not affect segmentation of the active material particles from the remaining phases, which is inherited from step one. In this study we chose "0-50" as the thresholding range to separate nano-pores from

the CBD (shown in Fig. S1g), in comparison to the cases of under-segmentation (0-40, 20% narrower, Fig. S1f) and over-segmentation (0-60, 20% wider, Fig. S1h) of this phase (pointed out by yellow arrows in each subset). The marker-based watershed is also added into comparison (Fig. S1i). Fig. S1j highlights the segmentation difference (white voxels) between the thresholding (as-used, 0-50, Fig. S1g) and the watershed method; an insignificant disparity is found. Fig. S1k displays the overview of the segmented pores in the data. Fig. S1l compares the porosity and tortuosity factor obtained by different thresholding range. It is found that the resultant porosity is linearly dependent on the thresholding range. The x-axis error bar represents the uncertainty range when choosing the thresholding range by the authors, resulting in a + 1.8% to -1.3% variation on porosity and + 0.4 to -0.8 variation on tortuosity factor, statistically insignificant as a consequence of the good quality and contrast of the data. In addition, the porosity and tortuosity obtained by marker-based watershed segmentation are shown as red and blue dashed lines, close to what we obtained using the thresholding method. Thus, it is reliable to use this thresholding method to separate the three phases using the workflow discussed above on this data.”

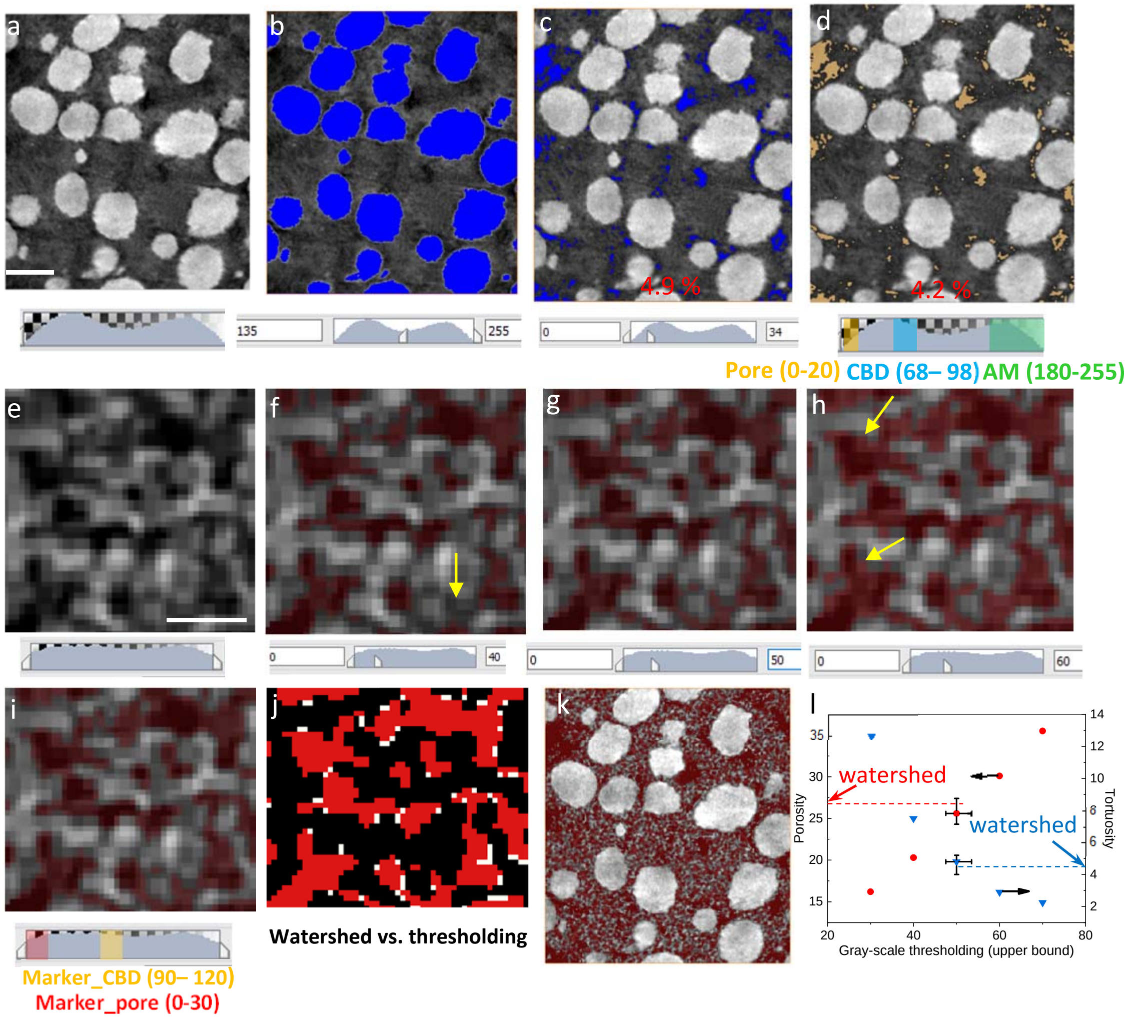

Fig. S1 Three-phase segmentation workflow using thresholding and the comparison with the marker-based watershed segmentation method (scalebar represents 7 µm in (a) and 2 µm in (e)).

2. Line 210-211, 454-455 and 38 in Supplemental: The authors implement dilute solution theory to

model the Li-ion transport. The Poisson-Nernst-Planck equations used in the present study result in different solution-phase potential drops than those derived from concentrated solution theory [1]. Concentrated solution theory is commonly used in Li-ion battery modeling, usually following the approach developed by Newman and colleagues. The paper must correct the citation in line 38 of the Supplemental material, which incorrectly references the Doyle-Fuller-Newman model as implementing dilute solution theory. Additionally, the authors found that the local discrepancy in electrolyte potential and charge-transfer current density are closely related (Lines: 216-217). Supporting text is needed in the paragraph (Lines 190-223) that discusses how using concentrated solution theory may affect the reported modeling results.

[1] A.M. Bizeray, D.A. Howey, and C.W. Monroe. Resolving a discrepancy in diffusion potentials with a case study for Li-ion batteries. J. Electrochem. Soc., 163 E223-E229 (2016).

The authors thank the reviewer for this point. Our Li-ion transport model comes from the generalised Poisson-Nernst-Planck (gPNP) equations arising from non-equilibrium thermodynamics as reported by Lai and Ciucci (Electrochim. Acta 56 (2011) 4369-4377, cited as REF[1] in the revised SI), who show that gNPN and Newman's concentrated solution transport models are "basically the same equations" (quoting Lai and Ciucci in Sec. 3.1 of their paper) upon substitutions of variables (reduced electrochemical potential of Li^+ for the solution potential) and parameters (ionic conductivity and ambipolar diffusivity, obtained from Eq. (18) and (19) in the SI).

Despite the similar equations, the main difference between the gPNP used in this study and the one referred as "diluted PNP" in the literature suggested by the reviewer (A.M. Bizeray, D.A. Howey, and C.W. Monroe. J. Electrochem. Soc., 163 E223-E229 (2016)) is that a concentration-dependent chemical diffusion coefficient D_tilde (Equation (8), (18) in the SI) and conductivity (Equation 19 in the SI) were used in this study, thus effectively considering non-unitary activity coefficients as in concentrated solution theory, being in contrast to the diluted PNP model in Bizeray et al.'s paper that uses constant self-diffusion coefficients.

As such, the gPNP equations we use are equivalent and provide as accurate results as Newman's (as well as Onsager-Stefan-Maxwell) concentrated solution theory, being remarkably different from dilute solution theory. We now refer "PNP" to "generalised PNP" to make a stronger distinction between concentrated solution and dilute solution theories.

The following are minor revisions that need to be addressed:
3. Line 3: The first author Xuekun Lu is associated with the Electrochemical Innovation Lab twice (i.e., there are two number ones above their name).

Thanks for pointing it out, now it has been correct it: now there is a letter "a" referring to both X. Lu and A. Bertei to declare that these two authors contributed equally to the work.

4. Line 111: What is the program/calculation used to extract the "volume-averaged tortuosity factor".

The tortuosity factor was measured in the commercial software Avizo, which is the same one as the thresholding segmentation, however, in case the readers intend to do similar study but have no access to Avizo, the authors have recommended an open-source software *Taufactor* in the *Methods* section.

5. Figure 6 d-e, g-h, and j-l need to state the C-rate and at what time the data is taken.

It was originally stated in the figure caption, but the authors have now added this in the corresponding context next to the figure thanks to the Reviewer's suggestion.

6. Lines 250-252 and 256-257: Particle size reduction (or morphological dilation) is nontrivial in a three-phase media. Additional text is required that discusses how the particle size is reduced near the separator "while maintaining the same particle volume fraction". Additional text is also required to support what the authors mean by "morphological dilation." The authors state that they shrink the CBD domain. Does this create more solid/electrolyte active lithiation sites?

The authors acknowledge that the details of morphological dilation/erosion and how to obtain the graded structure were not clearly described. Thus, the authors have added further complementary text as well as references in the manuscript next to Fig.6. Moreover, the authors also have added more technical details of this method and morphological dilation/erosion in *Methods* so that the flow of the manuscript is not affected.

For the second question, yes, improved mass transport and more solid/electrolyte interfaces are the two main advantages of high porosity. The authors thank the Reviewer for raising this point, thus further discussion about this point has been added in the paragraph above figure 7 as " It is noted that the rise of local porosity not only enhances the mass transport but is also accompanied with the increase of active particle/electrolyte interface. As the C-rate increases, the reaction active region resides closer to the separator, where R1 provides the most electrochemically-favourable conditions compared to R2 and R3."

7. Line 286-287: The authors state: "decreasing the CBD volume while maintaining the mass loading constant." The meaning of this sentence is unclear. To keep the mass loading constant did the authors increase the NMC volume fraction in this region? Do the authors instead mean that the NMC loading is held constant, while the CBD volume fraction is reduced?

While the pore phase was dilated, it is the CBD phase that was shrunk accordingly and the voxels constituting the active material were "locked" thus remaining unchanged. Further explanation has been added to make this clear in the paragraph below Fig. 7.

8. Figure 9k illustrates the porosity and tortuosity as a function of strain. How are these parameters calculated? Are they averaged over the entire domain or just the cut plane illustrated in Fig. 9d-f.

The authors segmented the 3D datasets scanned at different calendering strains and measured the porosity and tortuosity based on the 3D volume instead of the cut plane. This has been clarified now in the caption.

9. Line 437: The word "voxel" is oddly typeset.

The authors thank the Reviewer for pointing it out, and it has been corrected.

10. References: There are sometimes odd spaces before and after "– ". For example, see Line 487.

The authors appreciate this point and it has been corrected now.

11. Line 491 (References): There are dots where there should be dashes.

The authors have tried hard but unable to locate the issue indicated by the Reviewer. The names and paper title are correct. Should the paper be accepted, it will be fixed in theh production stage.

12. Figure S7: What is the x-axis?

It is the distance to the separator. It has now been added.

General thoughts:
13. The final section in the manuscript (Lines 330-381) seems tacked-on as opposed to a continuation of the paper. This section could be removed. Alternatively, the section could be strengthened by providing clear context relative to the body of the paper..

The authors appreciate the Reviewer's suggestion - the authors have added a new paragraph above the final section to highlight the link between the last section and the previous content.

14. The figures are not colorblind friendly. The same tone of red and green are commonly used in the same plot.

The authors have made most of the necessary changes of the colour scheme in the plots, thanks to the Reviewer's suggestion. Some of the figures, although the red and green are used, people still can distinguish them from the different symbols.

Reviewer #4 (Remarks to the Author):

This paper deals with some suggestions to improve the electrode design of NMC LIBs. To achieve this, a combination of visual 3D tomography technique, electrical measurement of coin cells and a physical chemical model in COMSOL was applied. To analyse the full structure of the NMC electrode a superimposition technique of high attenuation and low attenuation regions were overlaid. The model was used to identify better performances of the electrode while changing the ratio between pores and binder and applying graded particle sizes near the separator. Also the influence of calendaring of the NMC electrode is studied.
The final results of the paper regarding the calendaring for different cell types (high power / high energy) and the graded particle approach are relevant and presented well. However, the structure of the paper is very confusing. Some subsections may help to follow the different analysis and small connecting paragraphs between the sections would improve continuous comprehension and readability. The title is about 4D tomography, normally the fourth dimension in the literature is time, but here no time elapsed measurement is shown.

First Part: 3D characterisation

The author point out, that X-ray can be used to get insight of material structure. But there are some limits and a novel technique was used to overcome the limits.

• This novel technique is not explained in detail. If it is about using a short exposure time and a long exposure time and overlaying this images, it is not novel it is like HDR images. In addition, is this process before or after reconstruction? What is the meaning of high confidence in the context of this measurement?

As described in the second paragraph in the *Results* section, "a second X-ray nano-CT scan of the stand-alone CBD phase (i.e. no NMC particles) with a much higher voxel resolution (63 nm) was undertaken so that the micro-pores inside the blurry phase could be identified due to the enhanced signal-to-noise ratio and better spatial resolution and contrast". Scanning the electrode with long and short exposure time would not make much difference as it is the NMC particles that degrade the quality of the imaged CBD. Thus, we did a separate scan without NMC particles, just pure CBD, which significantly improved the resolution and contrast of the CBD phase. They were superimposed after reconstruction (text has been added in the manuscript to make it clearer). This technique is not the same as HDR, which actually has been used in X-ray imaging, known as dual-energy scan, on the same sample. By saying "high confidence", the authors intend to express "highly reliable/authentic" in comparison with other workarounds like inserting stochastically generated CBD phase into the microstructure. "high confidence" has now been deleted and replaced with "authentic CBD".

• Why didn't you use synchrotron X-ray to achieve a high contrast and resolution tomography?

Synchrotron facilities are not as easily accessible as the systems in the lab, as it requires a routine application process and proposal. The authors believe this study can benefit wide range of research groups who use lab-based CT systems in the universities. In addition, the Zeiss nano-CT the authors used in this study has a quasi-monochromatic, parallel beam, with the zone plates and capillary condenser, the contrast and resolution are very close to the state-of-the-art imaging beamlines like I13 at Diamond Light Source. For the scans conducted here, competitive spatial resolution and signal-to-noise ratio were obtained using a lab system, providing highly comparable data to synchrotron experiments.

• [p2; line 38] How is it possible to remove the NMC particles within a sample? If this is a physical process how do you ensure that it is not changing the structure?

We would like to take the opportunity to clarify the DSS technique. As the authors mentioned above, a pure CBD sample was scanned separately, instead of removing the NMC from the original sample. The pure CBD sample was fabricated using exactly the same procedure, as was explained in the first paragraph in *Methods*. The authors have added complementary texts in the paragraph to clarify.

• How can you be sure that the reconstructed data has no artefacts, void zones or noise? (compare 180° rotation vs. 360° rotation)

The authors need to clarify that experiment was conducted on a quasi-parallel beam system (Zeiss Xradia Ultra 810). For a full X-ray nano-CT scan the projection angle is from -90 to 90 degrees by default (instead of a 360 degree scan on cone beam system) and is reconstructed using a parallel beam filtered back projection; this is a standard, and widely adopted protocol. In addition, as the

nano-CT system operates with a parallel and using a pseudo-mono chromatic beam, there are minimal artefacts (see figure below) due to beam hardening, streaks or parallax errors, which are common in a polychromatic cone beam system and that is the reason why a full rotation (360°) is needed. The authors have optimised the sample preparation and scanning protocols in terms of sample thickness and exposure time (details in *Methods* section) to maximise signal-to-noise ratio. The robustness of the segmentation against noise is shown in the newly added Fig.S1 according to the later comment.

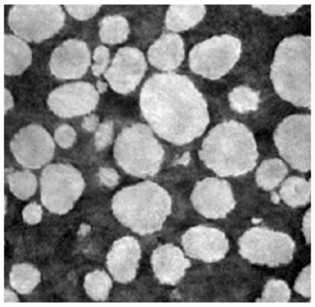

• More information about the reconstruction process is needed

The reconstruction process follows standard protocols, complementary texts have been added into *Methods* section as suggested by the Reviewer:

"The radiographic projections are automatically reference corrected, and centre shifts are aligned (post collection). The corrected, aligned radiographs are reconstructed using standard, parallel beam, filtered back projection algorithms, implemented in the Zeiss Scout and Scan software package (Carl Zeiss, CA, USA)."

Second Part: Microstructure and performance correlation
[p.5; line 4] Coin cells were prepared to show the correlation between model and experiment.
• Information about the coin cells experiment is missing (amount of measured coin cells, at which operating temperature). In addition, it is not clear if the model has the same material parameters as the used coin cell materials.

The complementary texts regarding the repetitions of the test (three) and at room temperature have been added in the *Methods* section. The electrode on which the scan was conducted and then modelled came from the same electrode sheet as the ones used for coin cell test, thus the material parameters are the same. More texts have been added in the third paragraph in *Methods* to emphasise this point. To complement this, the microstructure-resolved model does not require pre-defined material parameters such as porosity, active material volume fraction or size distribution etc, as they are resolved directly by CT, directly meshed and modelled, compared to homogenised model which uses these volume-averaged parameters to define the model.

Third Part: Design and optimisation of next-generation electrodes
Some subsections would improve the readability (procedure and steps, results and impacts and finally suggestions)
It is shown, that the shrinking of the CBD (or increasing the porosity) has a high impact of the electrical performance and the capacity.
• Hence the quality (noise) and threshold of the reconstructed data has even a big influence on the

results. This is not discussed and leads to a not trustworthy model. (250nm shrinking = 4 voxels = within measuring error?)

The authors understand the Reviewer's concern about the uncertainty and error originating from the data quality and thereby the thresholding segmentation method. Hence, following the Reviewer's suggestion, the authors have added a very detailed explanation and justification of the thresholding method used in this study (shown in Fig. S1 in SI).

The text related to it is copied below, some texts are also added in the second paragraph in *Results* section, and also in *Methods* section, pointing to the SI for the readers who are interested. 250 nm dilation of the pore structure was conducted based on the initial segmented volume (not grayscale data anymore), and controlled by automatic dilation algorithm (a detailed introduction of dilation/erosion is also added in the Methods section). In other words, the 250 nm dilation was not a consequence of measurement, but a set parameter for the dilation operation, so it is not related to measurement error.

"The gray-scale reconstructed data have clearly-defined boundaries between active materials and the other phases, which can be visualised in Fig. S1a and supported by the separated peaks in the histogram below it. Thus, the active material (bright phase) was firstly segmented using the thresholding range from the mid-point of two peaks (valley) to the high end to cover the second peak (Fig. S1b). However, the pore and CBD phases cannot be separated from the histogram, represented by the first peak on the left. Two reasons explain this: 1) the resolution is not high enough to resolve the nano-scale pores which are smeared with CBD; 2) even the macro-pores are darker in the data, but they cannot be reliably identified on the histogram due to the low volume fraction. However, at this step, it is still possible to segment the macro-pore from the remaining mixed phase due to its distinct contrast. An ideally-segmented macro-pore using the threshold value (0-34) (Fig. S1c) was compared with the marker-based watershed segmentation method [1] (Fig. S1d). This technique segments the data by growing from initially defined seeds in each region (the grayscale range defining the seeds in each phase is shown by the coloured overlay on the histogram), guided by the gradient of the gray-scale image, thus reliable boundaries between each phase can be obtained. It is found that the macro-pores segmented using two methods are very similar, visually judged by the segmented data and volume fraction (4.9% vs 4.2%).

The second step is segmenting the nano-pores after superimposing the stand-alone CBD scan with the original electrode. A higher magnification image of the CBD phase and the histogram after superimposition is shown in Fig. S1e. It is noted that the histogram is flattened, but this does not affect segmentation of the active material particles from the remaining phases, which is inherited from step one. In this study we chose "0-50" as the thresholding range to separate nano-pores from the CBD (shown in Fig. S1g), in comparison to the cases of under-segmentation (0-40, 20% narrower, Fig. S1f) and over-segmentation (0-60, 20% wider, Fig. S1h) of this phase (pointed out by yellow arrows in each subset). The marker-based watershed is also added into comparison (Fig. S1i). Fig. S1j highlights the segmentation difference (white voxels) between the thresholding (as-used, 0-50, Fig. S1g) and the watershed method; an insignificant disparity is found. Fig. S1k displays the overview of the segmented pores in the data. Fig. S1l compares the porosity and tortuosity factor obtained by different thresholding range. It is found that the resultant porosity is linearly dependent on the thresholding range. The x-axis error bar represents the uncertainty range when choosing the thresholding range by the authors, resulting in a + 1.8% to -1.3% variation on porosity and + 0.4 to -0.8 variation on tortuosity factor, statistically insignificant as a consequence of the good quality and contrast of the data. In addition, the porosity and tortuosity obtained by marker-based watershed segmentation are shown as red and blue dashed lines, close to what we obtained using the

thresholding method. Thus, it is reliable to use this thresholding method to separate the three phases using the workflow discussed above on this data.”

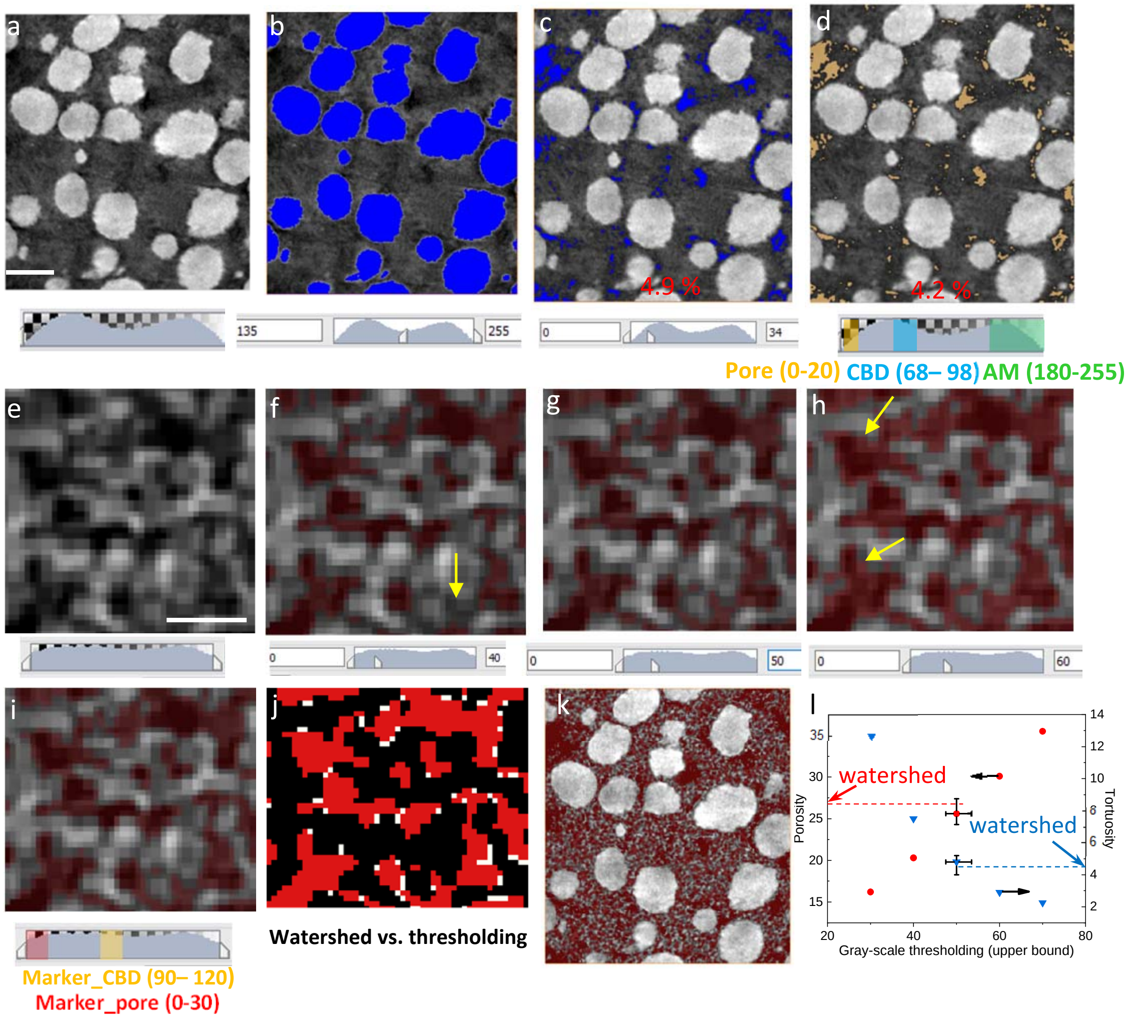

Fig. S1 Three-phase segmentation workflow using thresholding and the comparison with the marker-based watershed segmentation method (scalebar represents 7 µm in (a) and 2 µm in (e)).

• [p11. Line 29] This is the case in the most of BMSs

Thanks to the knowledge from the Reviewer. The authors have changed this from “is worth being integrated” to “which is often integrated in BMSs”

Fourth Part: Combining 4D X-ray nano-CT and electrochemical performance
This should be the main part related to the title.

• It is not explicit said, what is the fourth dimension? If its time, there are no experimental data or in-situ measurements.

“4D data” is an emerging terminology in the X-ray imaging field with a number of interpretations; to avoid confusion the authors propose to change the title to “3D Microstructure Design of Lithium-ion Battery Electrodes assisted by X-ray Nano-computed Tomography and Modelling”

Fifth Part: Conclusion

- [p.14 line 15] This is not a new but maybe a relevant insight.

The authors have changed the word “reveal” to “highlight” according to the Reviewer’s point.

REVIEWERS' COMMENTS:

Reviewer #1 (Remarks to the Author):

I read the revised manuscript and the response letter. The authors have addressed reviewer's comments and the manuscript should be published in Nature Communication.

Reviewer #2 (Remarks to the Author):

The authors have answered all points and revised the manuscript according to the reviewers' comments. Therefore, I think the manuscript is now sufficient to be published in Nature Communications.

Kang Dong

Reviewer #3 (Remarks to the Author):

The authors have considered carefully all our review comments and have revised the manuscript accordingly. This Reviewer recommends publication.

Reviewer #4 (Remarks to the Author):

All comments have been followed, the paper can be published.
